# On decoding of rapid motor imagery in a diverse population using a high-density NIRS device

**DOI:** 10.3389/fnrgo.2024.1355534

**Published:** 2024-03-11

**Authors:** Christian Kothe, Grant Hanada, Sean Mullen, Tim Mullen

**Affiliations:** Intheon, La Jolla, CA, United States

**Keywords:** fNIRS, BCI, high-density, motor imagery, diversity, demographics, bias

## Abstract

**Introduction:**

Functional near-infrared spectroscopy (fNIRS) aims to infer cognitive states such as the type of movement imagined by a study participant in a given trial using an optical method that can differentiate between oxygenation states of blood in the brain and thereby indirectly between neuronal activity levels. We present findings from an fNIRS study that aimed to test the applicability of a high-density (>3000 channels) NIRS device for use in short-duration (2 s) left/right hand motor imagery decoding in a diverse, but not explicitly balanced, subject population. A side aim was to assess relationships between data quality, self-reported demographic characteristics, and brain-computer interface (BCI) performance, with no subjects rejected from recruitment or analysis.

**Methods:**

BCI performance was quantified using several published methods, including subject-specific and subject-independent approaches, along with a high-density fNIRS decoder previously validated in a separate study.

**Results:**

We found that decoding of motor imagery on this population proved extremely challenging across all tested methods. Overall accuracy of the best-performing method (the high-density decoder) was 59.1 +/– 6.7% after excluding subjects where almost no optode-scalp contact was made over motor cortex and 54.7 +/– 7.6% when all recorded sessions were included. Deeper investigation revealed that signal quality, hemodynamic responses, and BCI performance were all strongly impacted by the hair phenotypical and demographic factors under investigation, with over half of variance in signal quality explained by demographic factors alone.

**Discussion:**

Our results contribute to the literature reporting on challenges in using current-generation NIRS devices on subjects with long, dense, dark, and less pliable hair types along with the resulting potential for bias. Our findings confirm the need for increased focus on these populations, accurate reporting of data rejection choices across subject intake, curation, and final analysis in general, and signal a need for NIRS optode designs better optimized for the general population to facilitate more robust and inclusive research outcomes.

## 1 Introduction

Functional near-infrared spectroscopy (fNIRS), first developed in seminal work by Jöbsis (1977) and Delpy et al. (1988), and others, is a brain imaging technique that uses near-infrared light sources and detectors (“optodes”) applied to the scalp to transmit light non-invasively through brain tissue and thereby monitor the relative abundance of oxygenated (HbO) and deoxygenated (HbR) hemoglobin species and changes thereof in response to functional brain dynamics. fNIRS thereby measures a similar effect to the blood oxygen level dependent (BOLD) brain responses known from functional magnetic resonance imaging (fMRI, see e.g., Logothetis et al., 2001), albeit with a much lower spatial resolution and a depth sensitivity that tends to be limited to 10–15 mm (Strangman et al., 2013). While many research-grade fNIRS systems are stationary instruments connected via fiber bundles to optode-fitted head caps, recent fiberless variants based on compact sources and detectors such as light emitting diodes (LED) and various types of photodiodes can be realized in wearable form factors that are by and large rivaled only by Electroencephalography (EEG), and whose costs are also decreasing as a function of improved miniaturization (Pinti et al., 2018; von Lühmann et al., 2021).

Non-invasive, compact, and potentially wearable brain imaging modalities are also of interest not only for basic neuroscience in freely moving subjects, but for the purpose of brain-computer interfacing (BCI). BCI as an engineering discipline has historically been focused on establishing communication and control channels for the severely disabled (e.g., Wolpaw et al., 2002) by leveraging intentional brain signals and interpreting them in real time as control outputs in home, care, and wheelchair settings, often relying on machine learning decoding of motor-cortex activity mediated by imagined movements (e.g., Pfurtscheller et al., 2003; Blankertz et al., 2006). While much of this work employs EEG, considerable research has also been conducted in fNIRS, using analogous motor imagery or execution processes, for example by Sitaram et al. (2007), Cui et al. (2010), Fazli et al. (2012), and Hosni et al. (2020). Such research efforts have collectively established motor imagery as one of few benchmark cases for machine-learning based brain signal decoding across both modalities, where a wide range of techniques have been tested in a similarly wide range of settings.

More recently, the spectrum of BCI applications has expanded to include uses cases outside intentional communication and control, for example in the form of passive BCIs (Zander and Kothe, 2011) that aim to supplement human-machine interactions with measures of ongoing cognitive state. This includes, for example, workload in air traffic controllers as studied by Ayaz et al. (2013) in an early case study in neuroergonomics, and more recent work by Pinti et al. (2015) in everyday tasks or Gateau et al. (2018) in pilots. Such use cases, enabled by relatively unobtrusive brain imaging, mark a gradual expansion of fNIRS out of the lab and into real-world contexts (von Lühmann et al., 2021), a process that mirrors a similar transition seen in EEG a few years earlier that has resulted not only in low-cost do-it-yourself EEG^1^ and now fNIRS^2^ kits aimed at the maker community, but in the case of EEG also consumer products ranging from simple toys such as the Mattel Mindflex^3^ to mindfulness-oriented headsets such as the Muse.^4^

However, so far, decoding accuracy has been a central challenge for non-invasive BCIs (e.g., Banville and Falk, 2016), fNIRS being no exception. While fNIRS has considerably lower spatial resolution than fMRI, an emerging class of high-density NIRS headsets aims to narrow this gap, suggesting that new levels of spatial imaging fidelity may be achievable in fNIRS while retaining wearability. In particular, the emerging field of high-density diffuse optical tomography (HD-DOT) leverages high-density devices, which typically measure at multiple source-detector distances and thereby produce depth-differentiated fNIRS readings (Wheelock et al., 2019). As the name implies, this approach marks a step from two-dimensional topography to three-dimensional tomography of brain dynamics, which promises, among others, improved spatial (depth) specificity in neural activation maps. Results such as those by Chitnis et al. (2016) have been encouraging and raise the question to what degree these imaging improvements have analogs in decoding capabilities in BCIs, which often similarly leverage differentiated spatial measurements using what is known in array signal processing as spatial filtering. This question led to the focus of this study on decoding from high-density NIRS devices.

With fNIRS maturing and its applications expanding, there is a need to address some of the remaining limitations of the modality, which may also aid overall decoding performance. Among them are the sensitivity to light obstructions from hair, particularly in the presence of relatively thick, dark, dense, or less pliable hair types (e.g., Orihuela-Espina et al., 2010), and the need to account for variable scalp properties such as reflectance and pigmentation (e.g., Fang et al., 2018). Some of these issues are exacerbated when measuring at scalp sites that tend to be covered with more hair (compared to, for instance, the forehead), as is the case in motor-control decoding. Such caveats are by no means unique to fNIRS (for instance, EEG faces its own set of hair- and skin conductivity related challenges, see e.g., Etienne et al., 2020), and there are ongoing efforts to mitigate these issues in fNIRS on the hardware side using innovative optode designs such as, for example, brush optodes (Khan et al., 2012). It may be assumed that these issues may present more in some headset and optode form factors than others, an aspect which is currently under-studied, and our study adds a data point featuring a state-of-the-art high-density device (LUMO, Gowerlabs).

In this light, we designed the present fNIRS study with a goal of not rejecting any participant due to hair phenotype, ethnicity, or other demographic criteria while faithfully reporting the impact of this choice on neural and BCI outcomes. To this end we collected a relatively large dataset of 61 sessions from 32 participants recruited from the general population primarily through an online ad (Craigslist), performing the same sequence of motor imagery tasks. We studied the hemodynamic responses associated with a hand motor imagery task in which subjects were prompted to imagine left- or right-hand finger tapping on a surface in response to a cue. However, unlike many prior fNIRS studies of this phenomenon (e.g., Abdelnour and Huppert, 2009; Cui et al., 2010; Yamada et al., 2012; Bak et al., 2019), we study a relatively short task performance period of 2 s, as opposed to the more common 10–20 s. This is motivated by the goal of higher information transfer rates in motor imagery applications such as cursor control or prosthetics but leads to a reduced signal-to-noise ratio (SNR).

As we will discuss in the following, in this challenging setup, degradations in signal quality can have a significant impact on results, and with it the effects of diverse hair phenotypes and demographic traits may become magnified. We present a panel of analyses to test for correlations between these traits and NIRS signal quality, machine learning performance, and neural responses that reveals several potential and previously known (e.g., Yücel et al., 2021), but rarely quantified statistical biases. At the same time, we note that our data sample is likely somewhat tilted toward university students, and the study uses a single (albeit state of the art) NIRS device form factor, which limits the scope of the study's findings. Factor levels are based on self-reported questionnaire data, which may limit sensitivity somewhat. However, we argue that the scenario studied here is quite relevant to fNIRS as it expands into new application areas (e.g., broad population studies, wearable/ambulatory settings, high-bandwidth BCI-based communication and control particularly from scalp sites other than the forehead, and a diversity of potential future consumer use cases), which underlines the need for both characterizing and addressing the root causes of NIRS signal quality degradations, and their effects.

## 2 Materials and methods

### 2.1 Experimental task

Our study is based on subjects performing a short-duration finger tapping motor imagery task over the course of three sessions spread out across several months (this time frame was significantly longer than originally planned due to the COVID-19 pandemic). In session 1, subjects performed an open-loop variant of the task, without BCI feedback. Subjects who returned for sessions 2 and 3 (all were invited but only about half returned for all sessions) performed a closed-loop variant of the same task that included feedback from a BCI model trained on the previous session(s).

**Figure 1:**
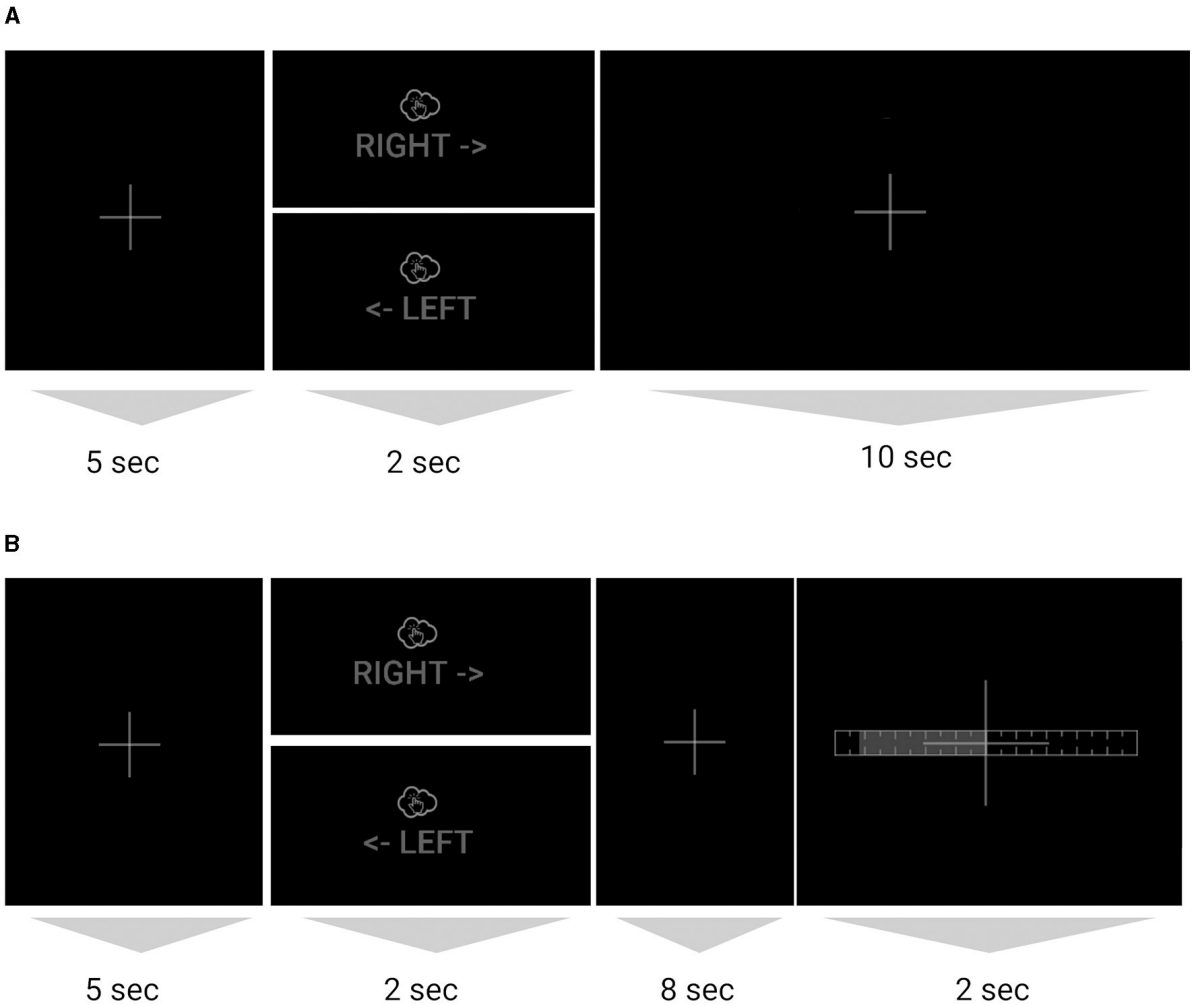
**(A)** Sequence of one trial with tapping (imagined or executed), for the open-loop session without BCI feedback and **(B)** for the closed-loop sessions with BCI feedback. The images are crops of the image/text shown on screen during that segment (full screenshots in Supplementary material). For each trial either the right or left hand image was shown. The dim color scheme reflects on-screen colors and served to minimize ambient light from the monitor.

Trial structure. The subject sat at a desk in front of a computer monitor with palms and fingers resting on the desk. The subject imagined a single tap with each of the index and middle fingers in succession (always in that order) at an instructed pace of ca. 1 Hz (once per second for each of the 2 s), with either the left or the right hand as indicated on the screen (Figure 1). Each trial lasted 17 s and consisted of 5 s of rest (fixation cross shown on screen), 2 s of imagined tapping (left/right hand indicated on screen for the tapping period), 8 s of rest (fixation cross shown on screen), and, in closed-loop sessions, 2 s of feedback during which the left/right prediction was shown using a horizontal bar indicating the confidence of the prediction, which reflected a single prediction and therefore did not animate. During the open-loop (i.e., no live BCI) session (Session 1), the fixation cross continued to be displayed for this duration (no feedback shown), and subjects perceived the resulting end-of-current-trial fixation period and beginning-of-next-trial fixation period as one contiguous 15 s fixation segment (except on the last trial).

Session structure. Each of the sessions was structured as a 2-min baseline measurement, followed by 4 blocks × 30 trials each, separated by 30-s breaks in between blocks, yielding 120 trials total (with equal numbers of left- and right-hand motor trials in a pseudo-randomized order). Each session was additionally preceded by a practice run of 30 open-loop trials (not included in analysis). During practice only, the rest periods in each trial were shortened and the word “tap” was flashed along with an auditory cue at 1 Hz beats, to help accustom the subject to the expected pacing of the taps. The total task time was ~40 min not including the setup time and practice block.

Special cases. The first session differed from Sessions 2 and 3 in that it periodically included a small number of trials in which subjects were asked to execute rather than imagine the tapping motion. These trials, which we do not analyze in this study, were designed to help reinforce the quality of imagery in the motor imagery trials and to reduce some of the monotony of the task in the absence of BCI feedback. To accommodate these trials, each block was divided into two sub-blocks each with 13 imagined trials followed by 2 motor execution trials. Therefore, Session 1 consisted of 16 motor execution trials and 104 motor imagery trials, while the remaining sessions had 120 motor imagery trials each. The sub-block type (“overt”/executed, or “covert”/imagined) and a reminder of the trial count was visually indicated on screen for 5 s before each sub-block. For consistency, this pre-block reminder was also displayed in Sessions 2 and 3 (always showing “covert”). See Supplementary material for uncropped task screenshots.

### 2.2 Data collection procedures

**Figure 2:**
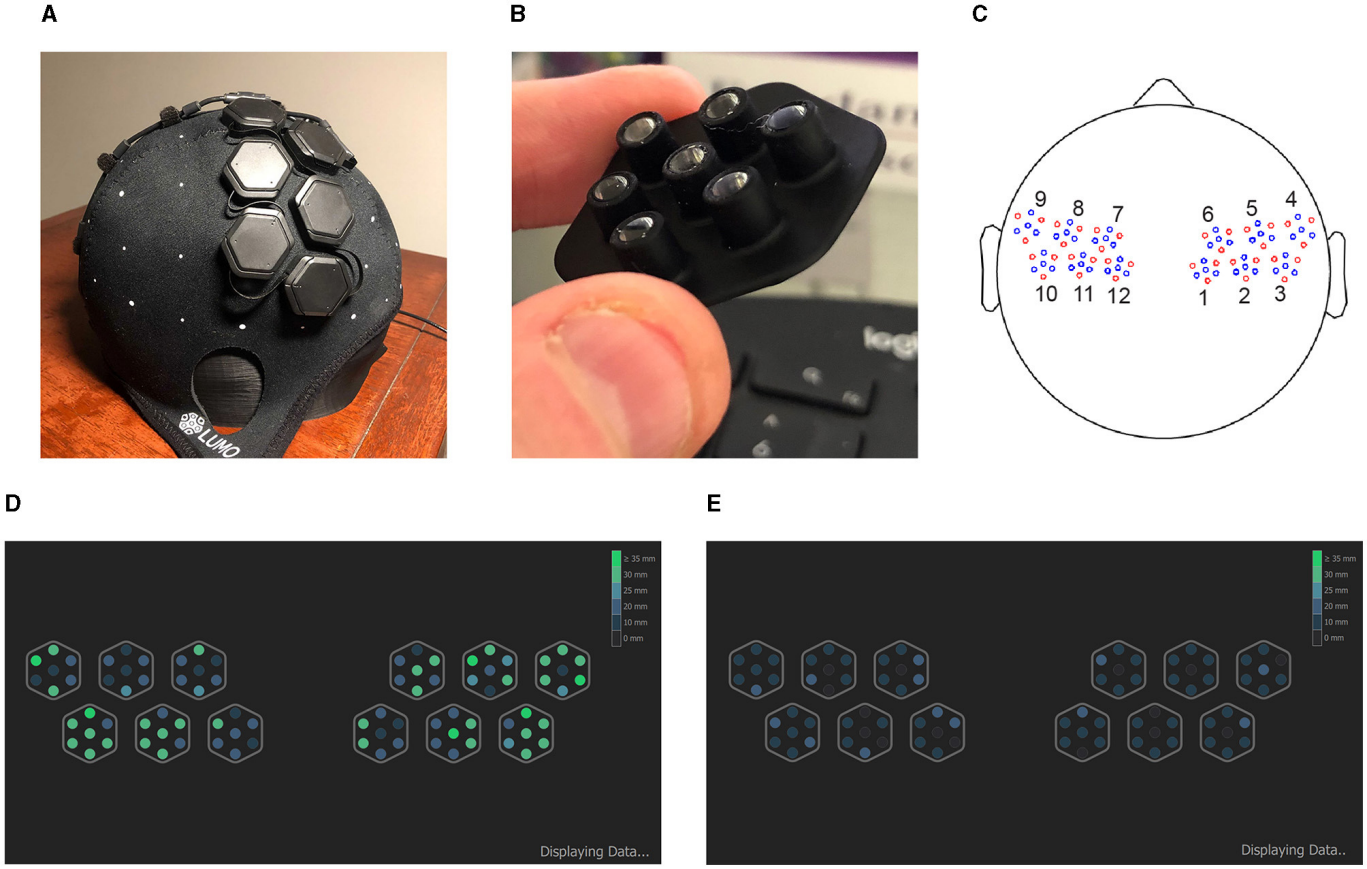
**(A)** A photograph of the LUMO NIRS headset used in this study, profile view. **(B)** A photograph of the light guide assembly for one of the tiles. **(C)** A montage of the same cap, top-down view, showing source optodes in red and detector optodes in blue, and a labeling of the 12 tiles. Bottom: Screenshot from the LUMO data acquisition application showing optode signal quality, for a typical “minimum viable quality” (MVQ) session **(D)**, and for a “poor quality” (PQ) session **(E)**. Optodes in green indicate the optode signal can be detected at a distance of 25 mm or more.

fNIRS data was collected using a LUMO headset from GowerLabs Ltd.,^5^ a wearable high-density NIRS headset with repositionable rigid tiles that each hold a set of 3 sources and 4 detectors (Figure 2 top row). The configuration used for this study featured 12 tiles, 6 on each hemisphere covering the motor and premotor cortex areas. The setup consisted of 36 dual-wavelength sources (735 nm and 850 nm) and 48 detectors, yielding 1728 dual-wavelength channels (3,456 in total across wavelengths) at a sampling rate of 10 Hz. The light guides used on the underside of the cap each were 5 mm in length and had 5 mm wide flat bottoms. The cap connects via a single wire to a hub which in turn was connected to a desktop computer via USB. We had two caps available, sizes 56 cm and 58 cm (head circumference).

Data from the LUMO device was captured using the LUMO software application from GowerLabs, which streams the fNIRS data over the Lab Streaming Layer (LSL) signal transport protocol (Kothe et al., 2024). This LSL data stream was in turn captured by the NeuroPype Experimenter Recorder software from Intheon^6^ and recorded to disk in XDF format.^7^ The latter also performed the task presentation. All software was run on a desktop computer running Windows 10.

Data was collected at the Swartz Center for Computational Neuroscience (SCCN) at the University of California San Diego (UCSD). The studies involving humans were approved by UC San Diego Institutional Review Board, La Jolla, United States under protocol number 140053. The studies were conducted in accordance with the local legislation and institutional requirements. The participants provided their written informed consent to participate in this study. The data was collected by an experienced research assistant who was trained in using the LUMO system as per the manufacturer guidelines and a backup research assistant trained by the former. COVID-19 protocols were followed as per SCCN and UCSD policies in effect at the time. Data collection took place in a small windowless air-conditioned room, with all overhead lights turned off. The screen monitor was set to a low brightness level, and a black background was used to further reduce ambient light sources (due to the low ambient brightness, on-screen contrast appeared higher than suggested by Figure 1). In Session 1, a dimmer lamp pointing at the ceiling was set to a low brightness to provide some ambient light and was turned off in Sessions 2 and 3.

At the beginning of each session, subjects filled out a questionnaire with basic demographics questions (age, sex, ethnicity), questions related to hair (length, color, density, strand thickness), and a question to rate their alertness on a scale from 1 to 10. Due to the multi-week gaps between the sessions resulting from delays caused by the COVID-19 pandemic, hair length and other properties did change across sessions for some subjects. Likewise, cap size was remeasured for each session to account for any such variability.

The cap was placed on the head by the experimenter and positioned in accordance with the vendor instruction manual, then adjusted multiple times as needed while the experimenter checked the signal quality in the LUMO application (Figure 2 bottom). Signal quality was indicated in the LUMO app by the maximum channel distance (in mm) at which a reasonable signal strength could be detected.

We found it a considerable challenge to obtain even a minimum threshold of signal quality with many subjects, particularly those with longer, thicker, and/or darker hair. The goal was to obtain a “good” (appear in green in the app, Figure 2 bottom) signal quality reading for at least one optode over the primary motor cortex on each hemisphere (4 innermost tiles on bottom row). However, if this was not possible after 20–30 min of attempted adjustments and recalibration, the session was collected regardless, in line with the study aim of collecting a diverse subject population capturing a wide range of hair properties without additional selection biases.

### 2.3 Dataset

Of a total of 33 subjects who recorded a Session 1, one subject had to be excluded due to a hardware disconnect issue in Session 1. All remaining 32 participants were asked to voluntarily return for Sessions 2 (15 did so) and Session 3 (14 did so). Due to the slow pace of recruitment due to COVID-19 policies during the pandemic and the summer semester at UCSD, there was a considerable gap, between 2 and 17 weeks across subjects, between Session 1 and Session 2, and a smaller gap of 2–5 weeks between Session 2 and Session 3. As a result, subjects' hair length, color, style and use of hair product may have varied between sessions, and for this reason, these properties are analyzed on a per-session basis. It can be expected that a “lockdown hair” effect (longer-than-usual hair) affected hair lengths in a few subjects, although it appears unlikely that the subject population has become unrepresentative as a result. Also due to the recruitment challenges, the final study sample was smaller than initially planned but was still sizable at 61 sessions. A summary of collected sessions by session number and demographics is shown in Table 1.

**Table 1:** Left panel: breakdown of subjects by session.

**Session #**	**Subjects**	**Excluded**	**Retained**	**Sex**	**Male**	**Female**		
1	33	1 (hw. err.)	32		16	16		
2	15	0	15^*^	**Ethnic Origin**	**White**	**Hispanic**	**Asian**	**Black**
3	14	0	14^**^		15	5	7	4
Total			61	**Age**	**18–25**	**25–40**	**41–55**	**>56**
					14	7	4	7

Right panel: breakdown of subjects by demographics. ^*^A subset of subjects from Session 1. ^**^A subset of subjects from Session 2.

### 2.4 Real-time BCI feedback

Sessions 2 and 3 included feedback from a real-time BCI to increase subject engagement and capture brain dynamics more representative of real-world closed-loop BCI interaction. The BCI used for feedback during data collection was a complex hybrid approach that leveraged data from both other subjects and prior sessions of the target subject and predates the methodology that is described in this article. The performance of this approach proved unsatisfactory and likely suffered from the same challenges alluded to in the introduction, yielding results in the 55–65% range across subjects. The method is described in detail in the Supplementary material.

This study instead focuses on a comprehensive panel of *post-hoc* analyses of the collected data using several previously published BCI methods, all of which had to be adapted to the dataset at hand due to the specific considerations in high channel density and rapid task timings. We focus our *post-hoc* analysis on the best-performing method, which was separately validated in Kothe et al. (2023, preprint under review at JNE), with adaptations noted in Section 2.7.2. This method requires no training data from the target subject, which enables calibration-free BCI usage, a desirable property for real-world deployment (e.g., Kindermans et al., 2014). This is accompanied by an analysis of the method's performance and other dependent variables in relation to physiological and demographic factors.

In brief, the training of the *original* hybrid BCI used for live feedback during data collection proceeded as follows. Data collection for the three sessions was performed in phases, where first all data for Session 1 was collected, and individualized BCI models were trained for each subject for use in Session 2. These BCIs used a transfer learning approach where a dimensionality reduction using the Common Spatial Pattern for Slow Cortical Potentials algorithm (Dornhege et al., 2003) was learned across sessions from all subjects to reduce the high-density fNIRS data to 300 spatial components. Then, for each subject, using that subject's Session-1 data only, a logistic regression-based classifier was trained on the reduced component space. For Session 3, the process was repeated after all Session 2 data was collected, with the difference that both Sessions 1 and 2 of a given subject were pooled for training.

### 2.5 Signal quality measures and session subsets

We measured signal fidelity using a panel of simple per-channel metrics, and a weighted whole-montage metric. The per-channel metrics were as follows:

- Coefficient of variation (Ayaz et al., 2010), for each wavelength (intensity), averaged across the session after discarding the baseline and break periods, computed from the mean and standard deviation for channel *c* as:

$$\begin{array}{l} {\text{CV}}_{c}=\;\frac{\sigma_{c}}{\mu_{c}} \end{array}$$

- A robust signal to noise ratio measure (in dB), for each wavelength (intensity), averaged across the session after discarding the baseline and break segments, computed as:

$$\begin{array}{l} {\text{SNR}}_{c}=10\log_{10}\frac{{\tilde{x}}_{c}}{\mathrm{median}|x_{c}-{\tilde{x}}_{c}|} \end{array}$$

where ${\tilde{x}}_{c}$ is the median of the channel's intensity signal.

- A stimulus-dependent signal to noise ratio measure as defined in Bak et al. (2019), separately for HbO and HbR as in:

$$\begin{array}{l} {\bar{\text{SNR}}}_{c}=10\log_{10}\frac{P_{\tilde{s}}\left(c\right)}{P_{ñ}\left(c\right)} \end{array}$$

See Bak et al. (2019) for a more detailed discussion of this measure. In brief, $P_{\tilde{s}}\left(c\right)$ is the optical density of the imagined tapping period (here 0 to 2 s relative to the onset of the MI stimulus), to which a Butterworth bandpass filter of 0.01 Hz to 0.1 Hz was applied and from which a baseline of the same duration immediately prior to the tapping period (−2 to 0 s relative to the onset of the MI stimulus) was subtracted; and where *P*_ñ_(*c*) is the unfiltered optical density of the same imagined tapping period (0 to 2 s relative to the onset of the MI stimulus).

Signal quality measures were computed and plotted using NeuroPype^8^ and NeuroScale Insights, both developed by Intheon (La Jolla, CA). NeuroPype itself relies in part on several open-source Python data science packages such as numpy (Harris et al., 2020), scipy (Virtanen et al., 2020), and statsmodels (Seabold and Perktold, 2010).

Besides channel-wise quality measures, we employed an overall session quality score to assess the quality of the montage as a whole, which was implemented as a weighted sum of per-channel coefficients of variation that places higher emphasis around the two left/right motor cortex regions of interest (ROIs). This ROI quality weighting was used since (1) with our high-channel montage it was neither practical nor optimal to have the experimenter spend the same effort on all channels during cap placement, particularly for difficult hair types, and (2) we hypothesized that BCI performance would be largely driven by the channels intersecting those ROIs. The weighted metric is normalized to 0 (worse) – 1 (best) and is specified in detail in the Supplementary material.

**Figure 3:**
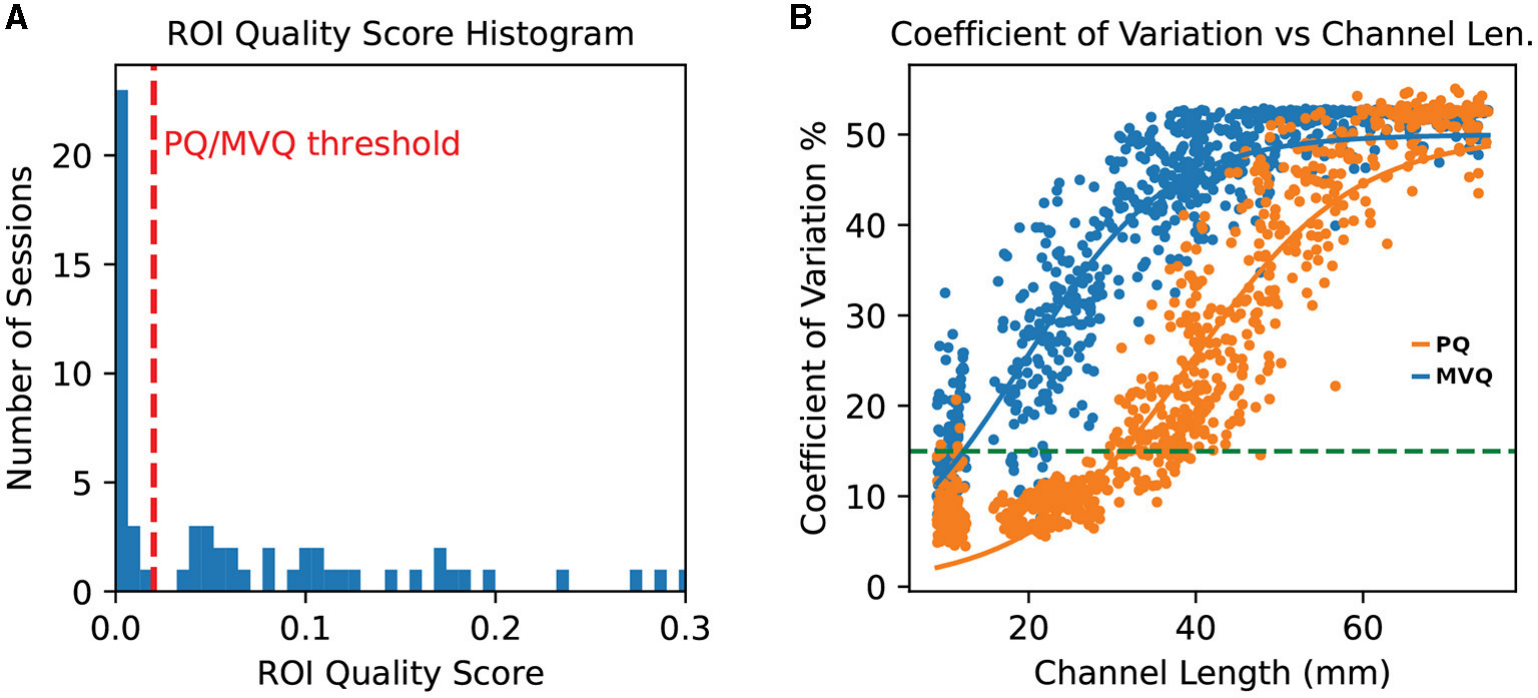
**(A)** histogram of ROI quality scores across all sessions. Note the cluster of numerous very low quality sessions on the far left. The *t*_qual_ = 0.02 threshold is shown as a vertical dashed line (red); note that thresholds as large as 0.03 would yield the same partitioning. **(B)** Per-channel grand average coefficient of variation, separately for poor quality (PQ, blue) and minimum viable quality (MVQ, orange) sessions, as a function of channel length. The horizontal dashed line (green) indicates the 15% CoV level. Channels above 75 mm were trimmed for greater legibility. Each dot represents one channel.

We found that this quality measure yields a compact cluster of lowest-quality sessions for our data (which we refer to here as “poor quality” or *PQ* sessions) that is separable from the quality of the remaining sessions. We refer to those *remaining* sessions as the “minimum viable quality” or *MVQ* sessions. We formally define these two sets of sessions, which are compared in subsequent analyses, in terms of a ROI quality threshold *t*_qual_, which we chose as the lowest upper bound of the lowest-quality cluster of quality scores (i.e., a highly conservative choice), which was for our dataset *t*_qual_ = 0.02 on the 0–1 session quality scale. A histogram of quality scores across all sessions along with the threshold is found in Figure 3 (left).

### 2.6 Within-tile averaging

To visualize the neural responses more easily and to limit the number of comparisons for certain statistics, the total number of channels needed to be significantly reduced in some parts of our analysis. For this we employed a within-tile averaging for each of the 12 optode tiles (6 on each hemisphere, labeled as in Figure 2C) in which the raw signal of all channels between the same two tiles were averaged together. This is equivalent to all source (or detector) optodes in a tile acting as a single multi-tip source (or detector) optode. For the following neural response analyses (block averages), we use this within-tile averaging and denote channels based on their source and detector tile numbers (e.g., Tile 1 to Tile 2 pairing written as “Tile1–2” in grid plots; numbering as in Figure 2C). Besides data reduction, this has the side effect of improving the effective SNR (e.g., coefficient of variation) of the newly formed “virtual” channels.

### 2.7 Block averaging

For visualization of observed fNIRS hemodynamic responses across subject groups, we follow a simple block averaging strategy of single-trial waveform segments. We did not employ Generalized Linear Model (GLM)-based statistical modeling (e.g., Yücel et al., 2021) here since task-related hemodynamic responses were separated by sufficiently long rest periods that they can be considered non-overlapping. To reduce the channel count and to increase the per-channel SNR, we first converted data to within-tile averages and then removed all resulting tile-to-tile (source-detector) channels whose source-detector separation (henceforth generally “length”) were outside a 15–60 mm range (here measured from center of tile to center of tile). This reduced the number of these (tile-averaged) channels to 44 (22 on each hemisphere).

We converted channel intensity for the two wavelengths to change in optical density relative to each channel's whole-session intensity average, followed by a series of common preprocessing steps: a bandpass filter at 0.02–2 Hz, Temporal Derivative Distribution Repair (TDDR) (Fishburn et al., 2019), and outlier trial removal using the Global Variance of Temporal Derivatives (GVTD) criterion (Sherafati et al., 2020) with threshold at 3 standard deviations. Short-channel regression was not employed at this stage for block averaging. We then estimated oxy- (HbO) and deoxy-hemoglobin (HbR) concentration changes according to the modified Beer-Lambert Law (MBLL) (Cope and Delpy, 1988) assuming a DPF of 6. Each imagined (“covert”) tapping trial was then epoched from 5 s prior to tapping onset to 17 s after onset, which ends just prior to the next stimulus presentation. For each trial, the signal average in the 2 s preceding the tapping onset was subtracted as a baseline measure, and all covert trials per session were averaged to obtain the block-average waveforms for all retained channels, separately for HbO and HbR and left/right conditions. Group analyses used 1-sample *t*-tests across sessions for each HbO/HbR concentration measure and were corrected for multiple comparisons over the time axis using the false discovery rate (FDR, Benjamini and Hochberg, 1995).

### 2.8 Machine learning methods

In the following we describe a collection of BCI approaches that we compare in an offline analysis of the previously collected study data. Specifically, we replicate a set of published BCI methods (both subject-independent and subject-specific), henceforth referred to as “reference methods.” All of these methods required some modifications since they were not designed for channel counts as high as in our data and/or task timings as short as in our design. We also test a recently developed method for very high-channel NIRS data described in Kothe et al. (2023) and note how it was adapted to this study. As this method performed best, we study its performance in greater detail in the following sections.

#### 2.8.1 Reference methods

We applied 5 published reference methods to our data, which we reimplemented using scikit-learn (Pedregosa et al., 2011) for machine learning and NeuroPype for signal processing to ensure sound implementations of the individual processing steps. We generally restricted ourselves here to (generalized) linear decoders, which currently represent the majority of published fNIRS decoder methods.

The compared methods roughly adhere to the conventional flow for brain-computer interfaces outlined in Mason and Birch (2003) and can be partitioned into preprocessing, a feature extraction stage, and a machine-learning stage, with methods differing in their choices at the respective stages. We briefly summarize these methods in the following with a focus on issues of dimensionality (e.g., channel counts) and processing and ignoring their task details; for complete details, the reader is encouraged to review the respective articles. Deviations from the originally proposed methods are also described in the following.

As is customary in fNIRS analysis, all methods apply a frequency filter to continuous data. However, to assess performance in a manner that is faithful to real-time usage, and for a fair comparison across methods, we generally ensured that all methods run *causally* by employing an approach modeled after the low-latency moving average convergence-divergence (MACD)-type (e.g., Gateau et al., 2018) bandpass filter by Cui et al. (2010) for all methods. To realize this, we first applied a high-pass filter using an exponential moving average (EMA) filter with a forget factor of 0.01 as in Cui et al., and subsequently a 2-s rectangular-window moving average lowpass filter. We found this to yield lower filter delay than the more common IIR-based high-pass filters, including low-order elliptic filters, with minimal impact on performance.

To tailor the methods to our short-duration trials, which was necessary since each method was built for different trial durations, we used the best subset of 3 per-channel handcrafted signal average features (2–4 s, 4.25–6.25 s, 6.5–7.5 s), which were originally developed in a previous pilot study using identical task timings and checked for agreement with hemodynamic responses on the present dataset. These time windows correspond roughly to what we identified as the likely “initial dip” (Hong and Zafar, 2018), followed by the first imagined tap, and the second imagined tap in our data (see also Figure 7 for analogous learned weight patterns in the high-channel model, which exhibit prominent peaks around those time windows). For methods that were designed for low-density montages *only* (all except Shin et al., 2017), we additionally employed tile averaging as a pre-processing step, since we found that these methods would otherwise overfit.

With these considerations in mind, we implemented subject-specific methods based on the following literature:

▸ We replicated a variant of the approach in Cui et al. (2010). In brief, the authors leverage a total of 48 NIRS channels over bilateral motor cortex at 10 Hz, which we assume to be of ca. 30–40 mm length; the authors rely on the amplifier software to derive optical density and ultimately concentration changes, and then apply the MACD-type bandpass filter as described above to this signal. The authors experimented with a number of different handcrafted features including amplitudes and gradients (slopes) but ultimately settled on the 11 most recent concentration values of the NIRS signal in a subset of channels selected using a criterion based on contrast-to-noise ratios (while always including both HbO and HbR for a given channel); their results show data from one participant where 10 channels performed best (yielding 10 × 2 × 11 = 220 features), although this would have differed across participants. They then apply a linear Support Vector Machine (SVM, Cortes and Vapnik, 1995) using a fixed cost value (*C* = 128) as the classifier. To replicate this method we first applied tile averaging to the raw intensity signal, which reduces the channel count to 348, and subsequently retained channels in the 25–45 mm range, which yields 36 channels in a similar length range as the original method. We then converted intensity to optical density following the MBLL and derived delta-optical density referenced to the first 60 s of the session so as to process all subsequent data in a causal manner. We then applied the aforementioned MACD-type bandpass filter, and estimated concentrations assuming a DPF of 6. We explored fine-grained temporal features similar to those used by Cui et al. (2010), but found that fewer longer-duration time averages performed better on our task with the linear SVM classifier, likely due to overfitting issues, and used the best subset of the aforementioned time-domain features, which amounted to all features. We tested a grouped F-score based channel selection but found mutual information based selection of individual features to perform somewhat better, and a variable feature count (using sequential feature selection) to perform somewhat worse, so here we retained the top-scoring 10 × 2 × 4 = 80 features, somewhat mirroring (Cui et al., 2010) example setup, using a mutual information criterion. For machine learning, we employed a linear C-SVM like the original authors but used a grid search to identify the optimal C parameter on the training data (which worked better on our dataset).
▸ We implemented a variant of the method in Shin et al. (2017). The authors utilize a device with a total of 204 channels at 8 Hz of three different channel lengths. They low-pass filter the raw intensity at 0.5 Hz using a 6th order non-causal Butterworth filter and reject channels where CV_c_ was greater or equal to 40 or the raw intensity was less or equal to 10 units. They then estimate concentration changes using the MBLL, and further bandpass filter that signal at 0.01–0.09 Hz with another non-causal 6th order Butterworth filter. For feature extraction, the authors use averages in three time windows (0–5, 5–10, 10–15 s following task onset); these features are of similar nature as ours but stretched over the longer task performance period of 10 s. The authors' method retains the best-performing two of the three available channel lengths, where 15 mm and 30 mm performed best by a small margin. For classification, the authors use two-level shrinkage Linear Discriminant Analysis (sLDA) where the first-level sLDA is applied separately to each of the two retained channel sets of same length, and the second sLDA takes in the two resulting output scores (i.e., a 2 d feature space). As before we replaced the non-causal bandpass filters by the aforementioned causal variant but use a 0.5 Hz Butterworth low-pass filter for purposes of coefficient of variation calculation, using the same threshold of 40 (a separate intensity threshold was not used since our setups' signal units are on a different scale). We retained channels of two length ranges (10–20 mm and 25–35 mm) *without* tile averaging, yielding 215 channels and thereby closely matching Shin's setup. For feature extraction, we used the three time window averages as noted above, again mirroring the authors' method relatively closely. For classification, we also employed a sLDA classifier, but using a single-level rather than a two-level classifier as a minor simplification (Ledoit and Wolf, 2004).
▸ A variant of the method outlined in Schudlo and Chau (2018) was also replicated. The authors use 22 NIRS channels of 30 mm length at 10 Hz and estimate HbO, HbR, and HbT concentration changes. They then low-pass filter these data using a 3rd order Type-II Chebyshev lowpass filter with a transition band of 0.1–0.5 Hz. The authors used visual inspection to remove specific artifactual channels, leaving between 12 and 17 channels across sessions. For feature extraction, the authors utilize a pool of per-channel slope features of different handcrafted time ranges out of which they select, using sequential forward search (SFS) a subset of the 4 best-performing features. They then trained a linear discriminant analysis classifier on the retained features. In our variant we first applied tile averaging to reduce the channel count to 348, and then retained channels in the 25–35 mm length range, which yielded 36 channels (times 2 wavelengths). We employed a coefficient of variation threshold (at 40) to remove bad channels (visual inspection would have been impractical given the larger channel and session count). As in the other methods, we replaced the IIR filter by the low-latency MACD bandpass filter (although we had experimented with the particular type of filter used by the authors in combination with a variety of methods in earlier analysis, but did not see enough of an improvement to justify the higher latency and deviation from the other methods). For feature extraction we used our predefined time windows across retained channels as the candidate feature pool and similarly implemented a SFS approach to retain the 4 best features. We then trained a plain LDA classifier on the resulting feature space.

We also tested subject-independent setups based on the following literature:

▸ We implemented a variant of the method in Trambaiolli et al. (2021). The authors record data from 32 NIRS channels at a sampling rate of 5.2 Hz and band-pass filtered the signal using a linear-phase FIR filter to 0.01–01 Hz and detrended the entire session (non-causally). They then calculated HbO and HbR concentration changes using the MBLL using a differential pathlength factor (DPF) of 7.25 and 6.38. They then extract a 30-s average feature per channel in a given trial. For machine learning they utilized an sLDA classifier with automatically determined shrinkage as implemented in the BCILAB toolbox (Kothe and Makeig, 2013). The authors implement an elaborate eigenvector-based feature selection to reduce the feature space. We replicated this method as follows: we first performed tile averaging to reduce the channel count again to 348 channels, and retained channels of 25–34 mm length, yielding 32 dual-wavelength features (i.e, similar to the prior study). We used the MACD-type bandpass filter in lieu of the FIR filter and skipped the detrending step to keep processing causal. For estimating concentrations we assumed here a DPF of 6 as in the other methods. For feature extraction we used here a single time-average feature spanning the interval of both taps (4.5–8.5 s) plus the initial-dip feature (which improved performance). We employ the same type of sLDA classifier, but used a more conventional F-score based feature selection method, determining the optimal feature count using nested cross-validation and searching over a range from 2 to 100.
▸ We also implemented a second variant of the method in Shin et al. (2017) (same as Shin et al., 2017 above) but applied to pooled sessions and optionally with causal zero-phase component analysis (ZCA) preprocessing (the ZCA approach is explained in more detail in the following section).

Subject-independent methods were evaluated as described in the subsequent Evaluation section, and subject-specific methods were evaluated using a 4-fold blockwise cross-validation *within-session*, adhering to the block structure of each session, in accordance with best practices recommended by Varoquaux et al. (2017).

#### 2.8.2 Subject-independent BCI

We additionally applied a generalized linear decoder for high-channel data that is closely related to that of Kothe et al. (2023), but which was tailored to the study at hand. While differences were relatively minor, we describe here the method in detail for the sake of completeness, noting differences in context. We retained all channels with a source-detector distance (“length”) of 50 mm or less and converted intensity to optical density using a log-transform (e.g., Hocke et al., 2018). At this stage, no whole-session time average was subtracted since processing was strictly causal. Instead, the previously described EMA high-pass filter was applied at this stage, as in the reference methods, yielding change in optical density relative to a running baseline.

Next, we decorrelated and standardized channels using a recursive ZCA (Bell and Sejnowski, 1996), an adaptive spatial filter that processes data here in 5-s block updates (this is applied causally and independently for each session); this step was also tested in combination with the Shin et al. (2017) method. Due to the high channel count, the underlying covariance matrix estimate was minimally regularized using shrinkage to prevent degenerate solutions (λ = 10^−6^ was found to be sufficient). This stage can be interpreted as a type of cross-session alignment or domain adaptation step, where sessions are spatially matched to have equal covariance [see also Huang et al. (2021) for a similar usage, and Kothe et al. (2023) for a motivation and more detailed presentation]. We found this to help with session-to-session transfer/generalization and with robustness to channels of poor SNR, which were prevalent in this study. Explicit bad-channel imputation as described in the original method was found to not improve performance on these data, and was not applied; likewise, removal of channels shorter than 10 mm and addition of an intensity bias were not applied for the same reason.

Next, we estimated HbO/HbR concentration changes using the modified Beer-Lambert law (assuming a differential pathlength factor of 6) (Delpy et al., 1988), also as in the reference methods. Lastly, for each motor imagery trial, we extracted a segment from 0 to 8.5 s relative to stimulus onset for subsequent classification and down-sampled each extracted trial segment to 5 Hz using polyphase resampling.^9^

We then robustly z-scored each of the resulting high-resolution spatio-temporal features using the training-set distribution for the respective feature (median and median absolute deviation) and applied a regularized logistic regression to obtain probabilistic single-trial predictions. We employ the subject-*independent* variant of the method, including the same Tikhonov-type spatio-temporal smoothness regularization, and also retaining the low-rank promoting regularization term acting on the spatio-temporal weight matrix **W**. This is the same idea also used for event-related potentials in EEG by Tomioka and Müller (2010) and can be interpreted as encouraging solutions where channels share linear combinations of few latent temporal weight profiles (time courses).

During training, the logistic regression coefficients are estimated by optimizing the following cost function, which is jointly convex:


$$\begin{array}{l} \min_{W,b}\frac{1}{N}\sum_{i=1}^{N}\log\left(1+e^{-y_{i}\left({\langle W,X_{i}\rangle }_{F}+b\right)}\right)+\alpha \left\|W\right\|*\\ \;+\beta {\left\|\Gamma_{U}\mathrm{vec}W\right\|}_{2}^{2}+\gamma {\left\|\Gamma_{V}\mathrm{vec}W\right\|}_{2}^{2}\; \end{array}$$

where ${\text{X}}_{i}\in {\mathbb{R}}^{C_{L}W\times T}$ is the matrix of z-scored chromophore features for the *i*'th trial, representing *W* wavelengths (here 2), *T* time points, *C*_*L*_ channels of length less than *L* (here *L* = 50 *mm*), while *y*_*i*_∈{0, 1} is the trial's class label. $\text{W}\in {\mathbb{R}}^{C_{L}W\times T}$ is the matrix of spatio-temporal weights, *b* is the bias, 〈·, ·〉_*F*_ denotes the Frobenius inner product, the matrices **Γ**_*U*_ and **Γ**_*V*_ are spatial and temporal Tikhonov operators, respectively, and $||\cdot |{|}_{{\;}^{*}}$ denotes the trace (or nuclear) norm. The parameters α, β, and γ are spatio-temporal regularization parameters that are learned from the data. The Tikhonov operators are formed as described in Kothe et al. (2023) using the same smoothing radius *r* of 15 mm and anisotropy factor of τ = 3/2.

We solve this optimization problem using an accelerated proximal gradient method (Beck and Teboulle, 2009), which converges in under 10 s on an Nvidia V100 GPU on our 59 or 60-session training sets (size depending on the subject) for a given set of regularization parameters.

#### 2.8.3 Evaluation

We evaluated the performance of the subject-independent models in a *leave-one-subject-out* cross-validation, where, for each session, we first train a model on all other sessions excluding any sessions of the target subject, and then test the model on that target session, on which we calculate average accuracy. We perform a one-sided *t*-test of all test sessions' average accuracy, against chance level (here 50% accuracy). Hyper-parameters governing the spatio-temporal regularization were optimized separately on each cross-validation fold's training set in a nested leave-one-subject-out cross-validation over α ∈ 0.025..0.075 and β ∈ 50..75, with γ fixed at 5. Note that these parameter ranges depend to some extent on the headset geometry and task timings, although wider ranges could be used at greater computational expense. The full nested cross-validation to assess performance of this model across all subjects was performed on a single-GPU machine on Amazon AWS in 2.5 days (since the method uses no per-subject training data, this is a one-time cost for this study, and the model does not need to be retrained for use with a new subject). We did not observe improvements from employing the *subject-specific* variant of the method, and we therefore restrict ourselves here to the subject-independent formulation.

### 2.9 Factor analyses

To examine pairwise correlations between BCI performance or ROI quality and subject factors (hair, demographics), we report three statistics: Pearson's correlation coefficient, Spearman's rank correlation coefficient (rho), and Kendall's rank correlation coefficient (tau). To keep the number of comparisons to a minimum, we limit our factor analysis to the best-performing method (the high-channel decoder). For additional insight our figures also show two trend lines, one fit with a least-squares linear regression, the other showing a robust fit using a Theil-Sen estimator (for visualization only). To examine correlations between binary-valued subject factors and BCI performance or ROI quality, we used a Wilcoxon rank-sum test as a robust analog to an unpaired *t*-test. Lastly, to compute the variance in ROI Quality and BCI Performance explained by subject factors (i.e., hair), we used a mass-univariate one-way ANOVA with FDR correction (Benjamini–Hochberg).

## 3 Results

### 3.1 Signal quality review

We reviewed channel quality, using the Coefficient of Variation (CoV) metric, as a function of channel length. Figure 3 (right) shows the group averages for the “minimum viable quality” (MVQ) and “poor quality” (PQ) sessions, where each dot represents the average across all sessions in that group for each probe channel (averaged across wavelengths). The figure shows that the sessions in the MVQ group follow a trend where nearly all channels up to about 30 mm length are of somewhat reasonable quality (though many still low) after which there is a nearly linear falloff in quality until settling into a limit around the 60 mm threshold. The overall progression is well explained by a sigmoid curve, and we show the best-fit curve in the figure. By contrast, for the PQ group, all channels above 25 mm have an excessive CoV (>15%) which rises approximately linearly from there, in a broad band, until converging with the MVQ group at the 60 mm range. This figure shows that the ROI Quality threshold used for binning of sessions into these two groups was effective, and the rejected (PQ) sessions have essentially no channel in the 25–45 mm range with an average CoV below 15%. We also found that all but one subject (1022) had either all of their sessions or none of their sessions scored as PQ, so the quality-based scoring was highly consistent across sessions of a given subject.

To allow for readable topographic quality plots given the LUMO's very large channel count, we averaged the raw channel metrics to the same between-tile resolution described in the Tile Averaging section, and excluded inter-hemispheric channels (which were all >60 mm).

**Figure 4:**
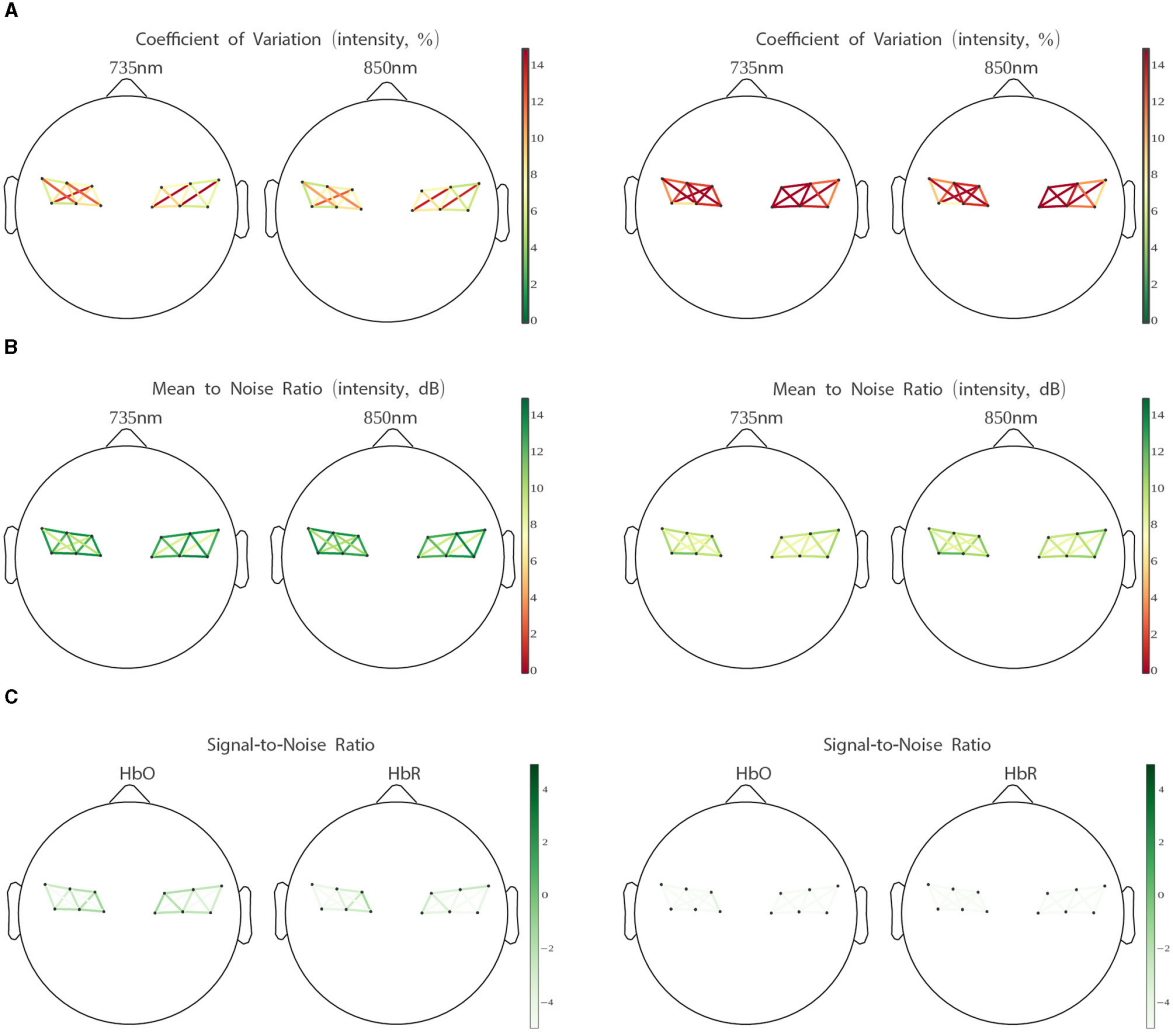
Coefficient of variation **(A)**, intensity signal to noise ratio **(B)**, optical density signal to noise ratio **(C)**, for “minimum viable quality” (MVQ) sessions **(left)** and “poor quality” (PQ) sessions **(right)**. Channels are averaged per LUMO tile (see Figure 2). See Section 2.3 (Channel Quality Measures) for explanations.

Also, to better characterize the quality of the signal data, we report our battery of channel quality measures separately for the MVQ and PQ session groups. The difference in quality between the two groups is clearly manifest in the group average plots in Figure 4, with the PQ group scoring very low on all metrics across all channels of interest.

### 3.2 Hemodynamic response averages

**Figure 5:**
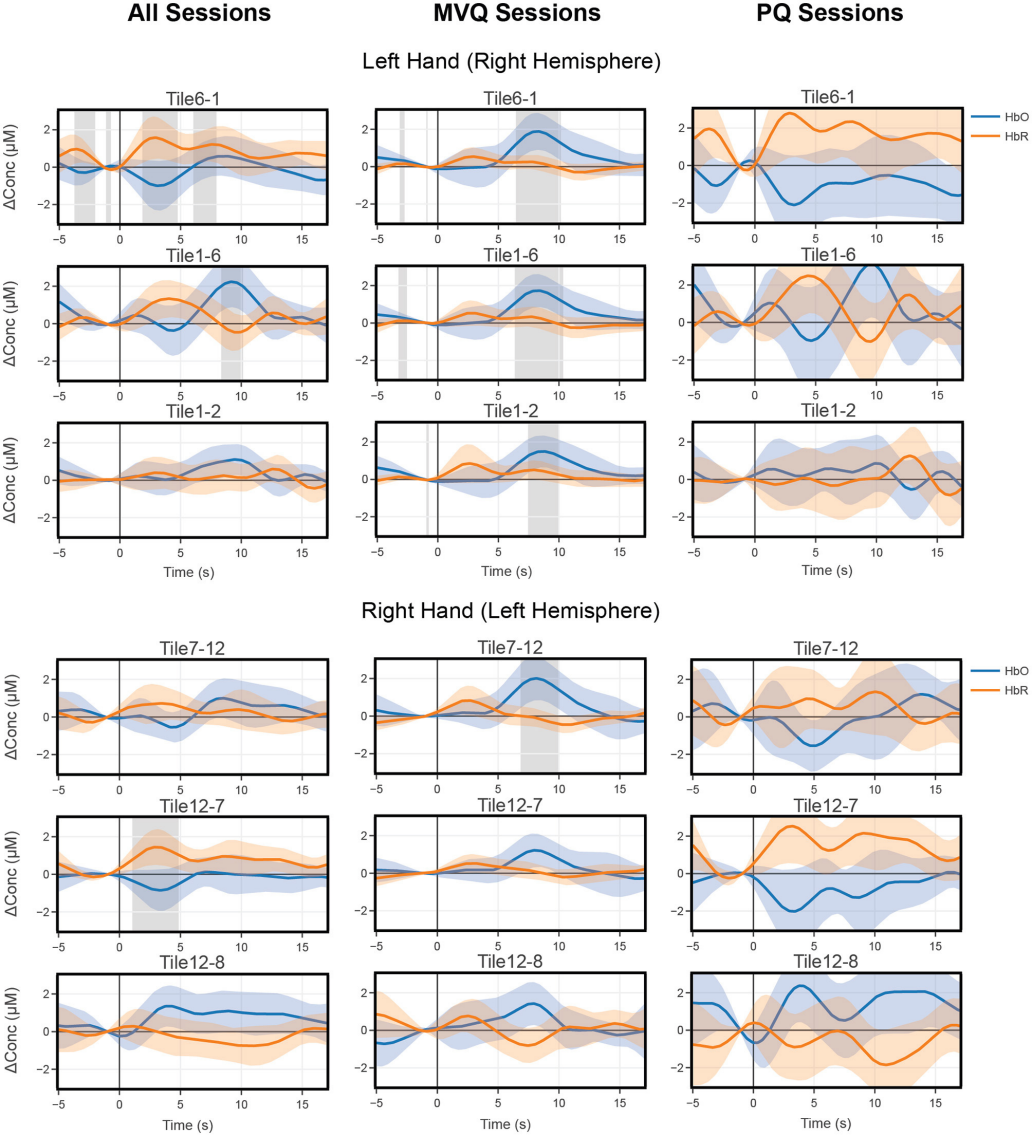
All **(left column)** vs. “minimum viable quality” MVQ (middle column) vs. “poor quality” PQ **(right column)** sessions mean block average concentrations of three representative right central motor area channels for 2 s imagery tapping (instructions presented at time point 0) with left hand for covert trials **(top three rows)** and of the left central motor area, showing right hand trials **(bottom three rows)**. Shaded error bars denote 95% confidence intervals. Gray bars indicate significant time periods (*p* < 0.05, FDR corrected).

Block average results were computed separately for all sessions (All), and the two quality-based session groupings described in Section 2.4, MVQ, and PQ. These are plotted side-by-side for comparison (Figure 5 top three rows) showing the same three representative contralateral right-central motor area channels (after within-tile averaging), here showing left-hand covert tapping. For these channels, the group of all pooled sessions (All) had *only one* channel (Tile1–6, which is situated approximately over left-hand motor cortex) with a significantly positive HbO peak near 9 s after onset (cf. Figure 2C for the tile indexing). A second channel (Tile6–1, i.e., with reversed source/detector optodes) showed a significantly positive HbR peak near 3 s, and a third channel (Tile1–2 also near left-hand motor cortex) showed a weak HbO activation which did not reach significance at any point. In contrast, the MVQ session group showed *all 3 channels* had a significantly positive HbO peak ~8 s after onset and the HbO concentrations showed stereotypical canonical hemodynamic response function (HRF) patterns with relatively low variability throughout the time course. HbR concentration also showed low variability but did not reach significance, though there was a slight concentration increase around 3 s after onset. The PQ group showed no significant concentration changes whatsoever for either HbO or HbR as there was high variability across the trials, and there was no clear indication of a canonical HRF waveform. One channel (Tile6–1) even showed an opposite effect with negative HbO peaks and decreased levels of concentrations across the trials.

Similarly, for right-hand motor imagery we show three representative contralateral left-central motor area channels (after within-tile averaging) for comparison between these session groups. Here, only one channel (Tile7–12, over right-hand motor cortex) in the MVQ group shows a significant HbO peak around 8 s. The remaining channels across all other groups show no significant time periods, but the patterns shown in left hand tapping are also present here, with the MVQ group showing stereotypical canonical HRF waveforms with HbO concentration peaking around 8 s. Although the relative concentration levels were about the same as the left-hand tapping condition, there was overall higher variability near the peaks. Both the PQ group and all group show noisier waveforms and higher variability lacking the expected canonical HRF waveform patterns.

### 3.3 Machine learning analysis

#### 3.3.1 Reference methods

Table 2 compares the overall performance across all tested methods, using the MVQ group of sessions. None of the selected reference methods appeared sufficiently well adapted to channel counts as high as that provided by the LUMO, so none are likely optimal for our dataset. Nevertheless, we made a concerted effort to adapt parameters (e.g., feature timings and channel reductions) within reason. As the table shows, all reference methods struggled to reach adequate performance on our challenging dataset, with performance in the 50%−60% range. Significance levels from one-sided *t*-tests vs. the chance level of 50% are indicated in brackets (uncorrected).

**Table 2:** Performance comparison of different BCI methods on study dataset (MVQ session group).

**Method**	**Accuracy**
**Subject-Specific**	
Cui et al. (2010)	57.3 +/– 11.8% (^**^)
Shin et al. (2017)	54.5 +/– 12.3% (n.s.)
Schudlo and Chau (2018)	53.3 +/– 11.5% (n.s.)
**Subject-independent**	
Kothe et al. (2023)	**59.1** **+/– 6.7%** (^**^)
Shin et al. (2017) (with ZCA)	54.1 +/– 5.0% (^**^)
Trambaiolli et al. (2021)	53.5 +/– 6.4% (^**^)
Shin et al. (2017) (no ZCA)	51.8 +/– 6.3% (n.s.)

Outcome of a one-sided t-test vs. chance level (50%), here based on uncorrected p-values, is indicated in brackets using ^*^ for significant (p < 0.05) and ^**^ for highly significant (p < 0.01) outcomes.

The performance of the high-channel decoder can be interpreted as roughly doubling the gap to chance level (here 50%) compared to most of the other tested methods, except Cui et al. (2010). However, we note that the goal of this comparison is not to benchmark any specific published method, but to upper-bound the overall performance achievable with various published state-of-the-art fNIRS approaches on the collected dataset.

#### 3.3.2 Subject-independent method

In the following we review individual-session results across all collected sessions using the best-performing method. We performed a leave-one-subject-out cross-validation (LOSO CV), where all sessions of a given test subject are excluded from the respective training set, representing performance in the subject-*independent* case where no individualized training data is needed for BCI usage. We report performance averages at the level of individual sessions here, since over half of the subjects participated in only a subset of the 3 sessions.

**Figure 6:**
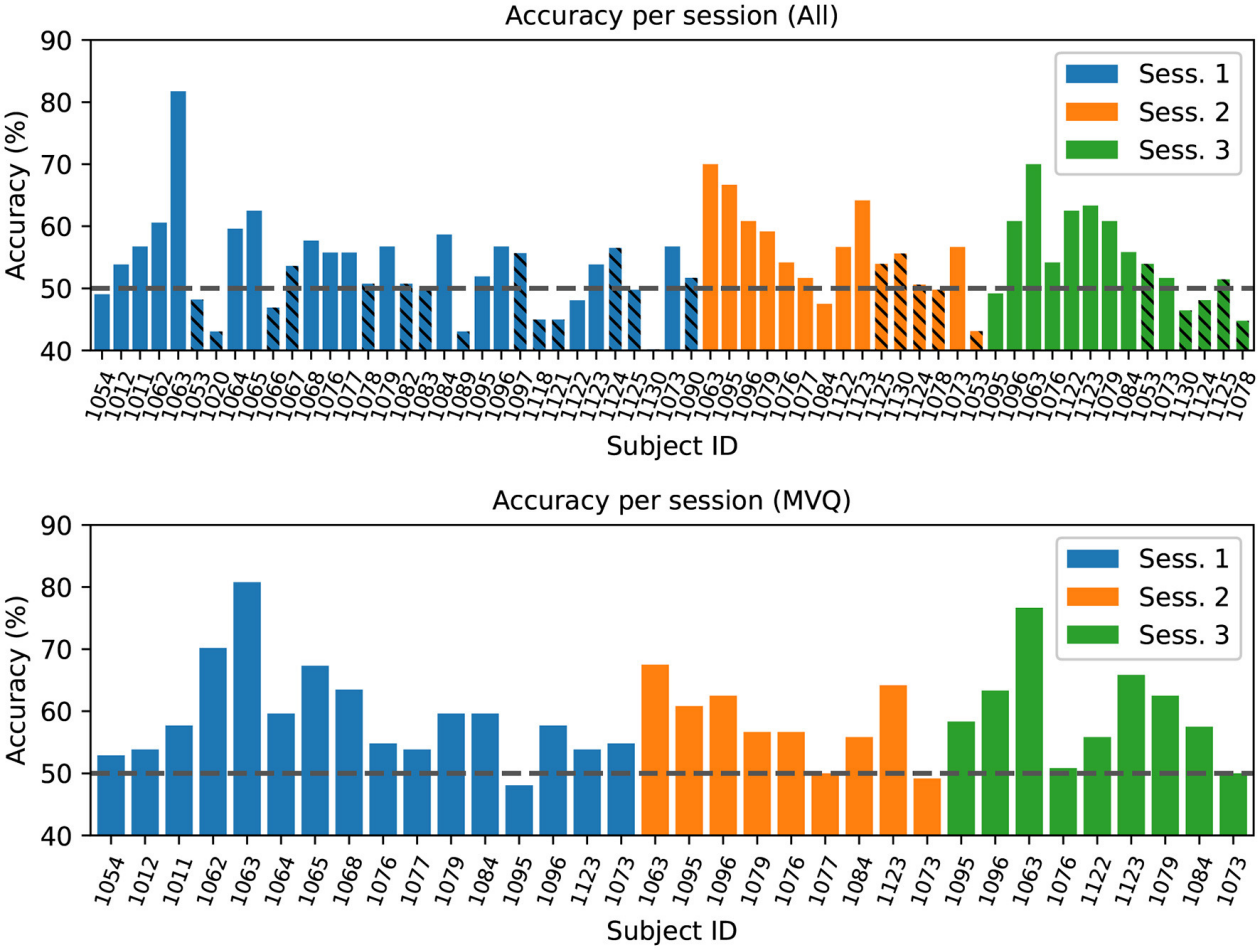
**Top**: Leave-one-subject-out cross-validation accuracy, training on all other subjects' sessions in the dataset, results grouped/colored by session number (1–3). The cross-hatched sessions were identified as “poor quality” (PQ) based on a ROI quality index < 0.02. Bars are ordered by data collection time and by session. **Bottom**: Cross-validation results for model trained on the subset of sessions that had at least minimal quality. The chance-level accuracy (50%) is indicated by the horizontal dashed line.

We performed two runs of this analysis: (1) over all sessions (All), and (2) over the previously defined MVQ session group. Figure 6 (top) shows results from the all-sessions cross-validation. This analysis confirms that the majority of sessions with poor data quality (the PQ group discussed earlier, shown cross-hatched) tend to have about chance accuracy as one might expect, with only a few exceptions. Average accuracy across all sessions (including PQ, Figure 6 bottom) was near the 50% chance at 54.7 +/– 7.6% (however, sig. at *p* < 0.05 in a one-sided t-test vs. chance). A leave-one-subject-out CV run on the MVQ sessions yielded an accuracy of 59.1 +/– 6.7% (significantly different at *p* < 0.01). One might assume that including additional subjects in the training set (i.e., adding the PQ group to the training but not test set) may aid performance on the MVQ data; however, this was *not* the case, and doing so resulted in a performance degradation. Average performance on the PQ group is at chance with 49.9 +/– 5.3% (n.s., despite including MVQ sessions in the training set to help the learning).

#### 3.3.3 Spatio-temporal model weights

**Figure 7:**
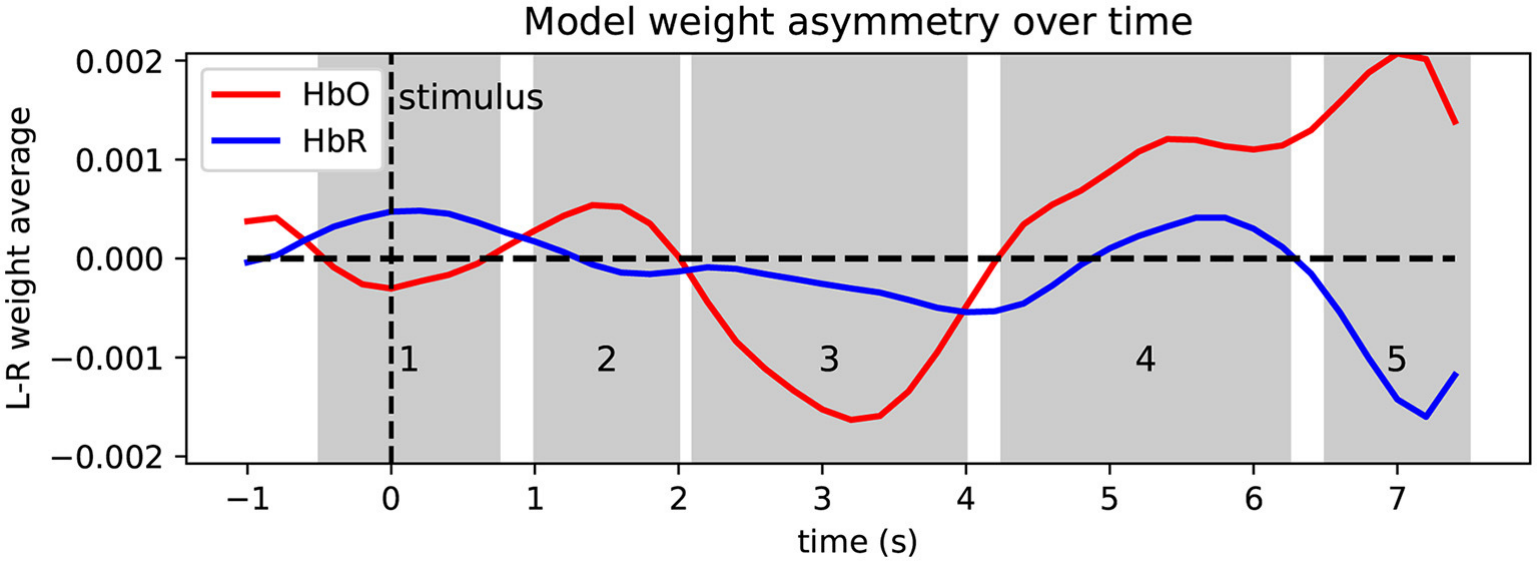
Temporal evolution of model weights relative to stimulus onset, showing weight asymmetry (left minus right difference) separately for HbO (red) and HbR (blue) features. The time axis is re-labeled to account for the 1 s moving average filter lag that precedes the ML stage; if filter lag was ignored, the stimulus onset (*t* = 0), would be at the −1 marking. Shaded regions 1–5 indicate time windows for which average spatial weight is plotted (subsequent figure).

**Figure 8:**
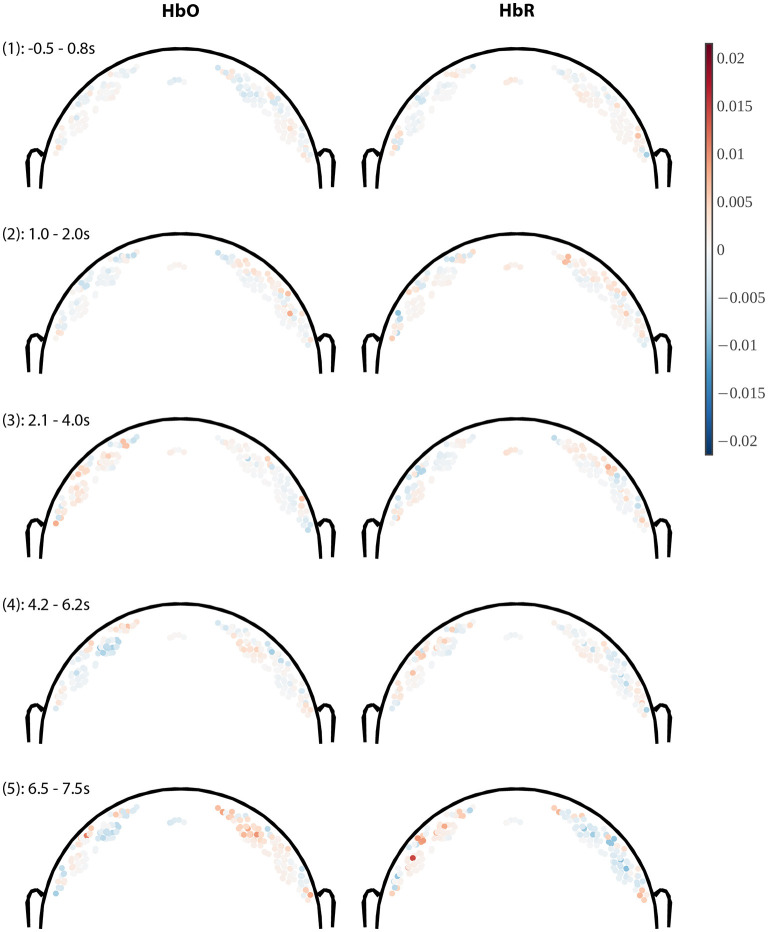
Coronal slices through model weight distributions averaged within 5 variable-length time windows. Only channels whose midpoint lies within +/– 2 cm of the coronal plane (through Cz) are plotted, where the hue of the dot indicates weight polarity (red = positive, blue = negative) and saturation indicates weight magnitude (all images use the same color scale). Channels are plotted at a depth proportional to the channel length (length/4). Weights for HbO-derived features are shown in the left column and HbR on the right. Panel headings indicate the time window (1–5) and the time range over which the weight was averaged in seconds (cf. Figure 7).

To aid interpretation of the learned patterns and as a visual sanity check, Figures 7, 8 depict the model weights learned on the MVQ session group in time and space. We again restrict ourselves to the best-performing model. Figure 7 shows a summary of the model weights' temporal evolution over the course of the portion of the trial used for prediction, where we show the *averaged* model weight over left-hemisphere channels minus average right-hemisphere channel weight at a given time point (i.e., overall asymmetry in model weights as it varies over the course of a trial), separately for HbO and HbR. The time courses exhibit several peaks of varying polarity, which suggests several change points and temporal phases in the brain dynamics leveraged by the model. We identify 5 phases (shaded regions labeled 1–5 in the figure), for each of which we display the spatial weight distribution, averaged within the respective time window (cf. Figure 8). To further aid interpretation, the time axis is relabeled to correct for the effective delay introduced by the causal preprocessing pipeline (1 s), so 0 s (dashed line) shows weights corresponding to the stimulus onset. Shaded regions 4 and 5 likely correspond to the HRF peak induced by the 2-s imagined tapping task. The large negative HbO deflection in region 3 appears most compatible with the timing and magnitude of what has been identified as an initial dip of the hemodynamic response [e.g., described in Hong and Zafar (2018) and references therein]. Regions 1 and 2 resemble weight “swings” flanking the highest-weighted features that are often seen in learned linear filter weights, and which could be involved in noise cancellation.

In Figure 8 we show the model weights for channels near the coronal plane (within +/−2 cm from the plane) in coronal slice plots viewed from the back of the head (nose pointing away from the viewer). Longer channels are plotted deeper in line with a simplified NIRS photon path model (e.g., Strangman et al., 2013) for the purpose of visualization. A channel is then rendered as a single-colored dot at the linear midpoint between source and detector, offset toward the head center by a distance proportional to the channel length (using the formula for *mid*_*c*_ given in the Supplementary material with proportionality factor τ = 4).

Briefly, we can see that absolute weights increase toward later time windows, and that within each time window the spatial weight distribution is relatively smooth, which is expected due to the spatial regularization. The weight gradient is relatively steep between the short (shallow) and longer (deeper) channels, which is visible as a polarity reversal in most plots that happens between depths differing by no more than 0.5–1 cm, which is compatible with the model performing implicit short-channel regression. Across time windows we can see prominent weight hotspots about 2–3 cm diameter near the hand motor areas in the last and second-to-last time windows with opposite polarities for HbO/HbR, in agreement with a physiological origin. Time window #3 (2.1–4 s) remarkably shows nearly the opposite HbO effects as the subsequent windows, again compatible with the effect described as an initial dip in Hong and Zafar (2018).

In summary, these results confirm that the model learns brain dynamics that are overall compatible with a short-duration left/right hand motor imagery task.

### 3.4 Factor analysis

#### 3.4.1 ROI quality vs. hair and demographics factors

**Figure 9:**
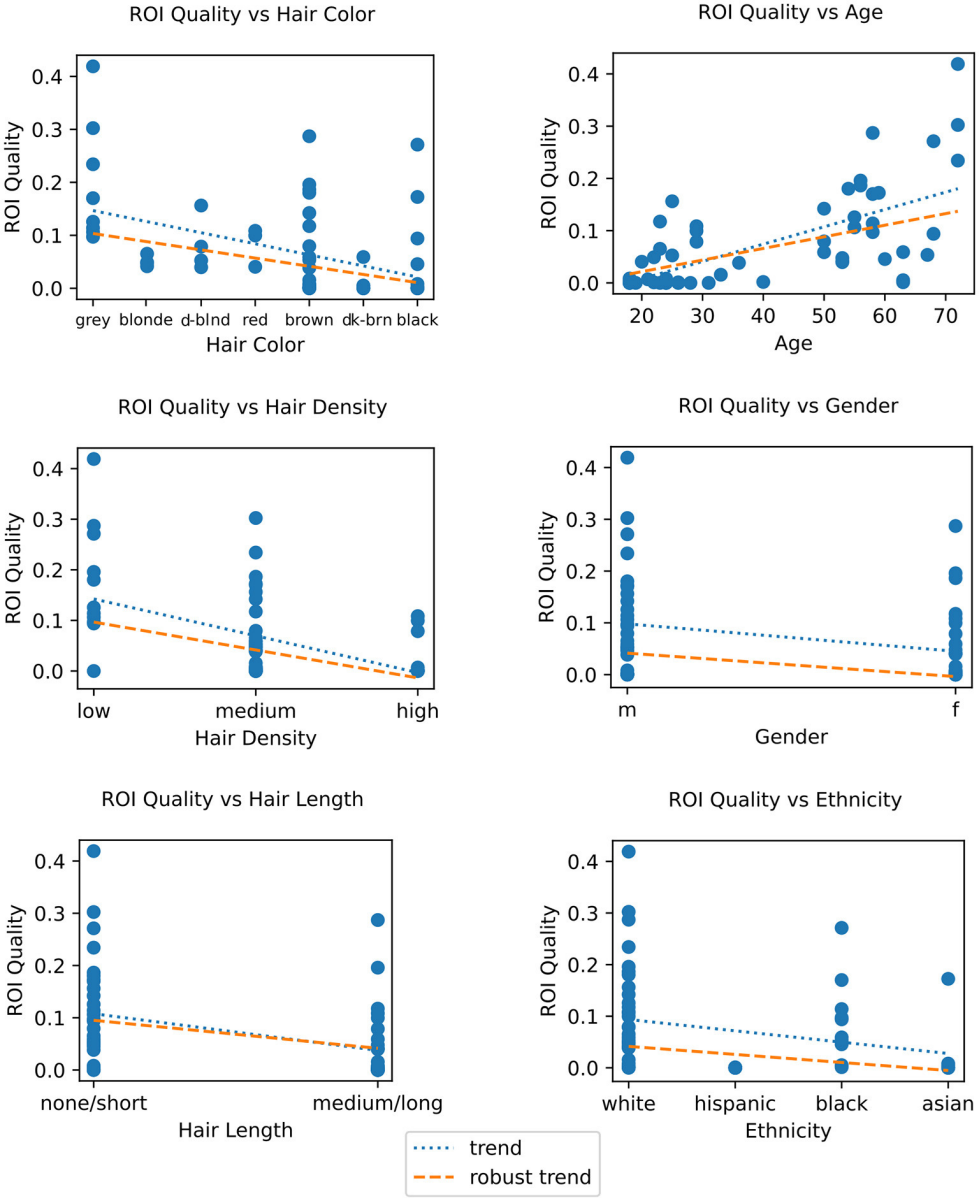
**Left column**: ROI quality vs. demographic traits, showing age **(top)**, sex **(center)**, and ethnicity **(bottom)**. **Right column**: ROI quality vs. hair traits, showing color **(top)**, density **(center)** and length **(bottom)**. For ordinal or continuous quantities, Pearson's correlation coefficient, Spearman's rho, and Kendall's tau are provided, and for binary quantities, a Wilcoxon rank-sum unpaired *t*-test statistic is shown. Each dot represents one session. Fitted trend is shown in dotted blue, and robust trend (Theil-Sen estimate) is shown in orange. For FDR-corrected statistics, see Table 3.

To better understand the factors contributing to low signal quality, and in turn generally low BCI performance, we first computed the correlations between subject factors impacting signal quality, primarily those related to hair, and the ROI quality score described in Section 2.4. For categorical factors with only two values, an unpaired Wilcoxon rank-sum test was used, while for factors with more than two values the Pearson correlation coefficient was computed along with a robust Kendall's T statistic (tau), and Spearman's rho. These all show statistically significant correlations between the ROI quality score and the color, length, or density of the subject's hair (see Figure 9 and Table 3). We found the robust correlation measures to be somewhat more conservative, and report these in the following text, after FDR correction for multiple comparisons. No other comparisons were performed beyond those reported in this section.

**Table 3:** Top: ROI quality variance explained by hair length, hair color and hair density, computed using a mass-univariate one-way ANOVA with FDR correction, along with the sum of squares, mean of squares, F-stat, and *p*-values.

**ROI quality**	**Variance explained**	**Sum of squares**	**F-statistic**	***p*-value (FDR)**
Hair length	16.14%	0.0835	15.8410	0.0002 (^**^)
Hair color	14.21%	0.0735	13.9432	0.0004 (^**^)
Hair density	8.51%	0.0450	8.3479	0.0054 (^**^)
Residual	61.14%	0.3162	n/a	n/a
Ethnicity	16.72%	0.0866	7.7657	0.0002 (^**^)
Sex	6.96%	0.0360	9.6949	0.0029 (^**^)
Age	34.69%	0.1794	48.3405	0.0000 (^**^)
Residual	41.63%	0.2153	n/a	n/a

Bottom: ROI quality variance explained by ethnicity, sex and age, computed using a mass-univariate one-way ANOVA with FDR correction, along with the sum of squares, F-statistic and p-values. ^*^ and ^**^: Significance levels (thresholds as in Table 2).

For hair length we combined subjects' “none” and “short” responses into one category and “medium” and “long” into a second category due to too few “none” and “medium” responses, and performed an unpaired robust Wilcoxon rank-sum test. The result, as shown in Figure 9 (bottom right), was a statistically significant association where medium or long hair predicted lower ROI quality (*T* = 3.347, *p* = 0.003).

A look at hair density and color (Figure 9 right) showed greater hair density to be significantly negatively correlated with ROI Quality (tau = −0.325, *p* < 0.001). Likewise, when comparing ROI quality with hair color, we again find a significant correlation (tau = −0.399, *p* = 0.001), with darker hair resulting in lower ROI quality. Hair color was rank-coded based on a sorting of self-reported hair color by approximately lightness (see Figure 9). Our results concur with findings in other studies where hair density and color were found to be inversely correlated with signal quality (Khan et al., 2012). We also looked at hair strand thickness as self-reported by subjects, but found it unusable as most subjects reported it as simply “medium.”

Since hair color, density and length often vary between ethnicities, genders, and ages, we examined the relationship between these three factors and the ROI quality score. We found a significant positive correlation (tau = 0.486, *p* < 0.001) between age and ROI quality, as shown in Figure 9 (left top), with subjects over 50, who are likely to have thinner and greyer hair, having a significantly higher ROI quality score than subjects under 30. As for sex (gender at birth), an unpaired Wilcoxon rank-sum test showed that male subjects had a statistically significant higher ROI quality score than female subjects (*T* = 2.279, *p* = 0.025) (see Figure 9 left center.). Lastly, binned into broad ethnic categories for the sake of a correlation analysis (and due to the relatively small sample size, see Table 2), we observed a highly significant correlation between the ROI quality score and ethnic origin (tau = −0.391, *p* = 0.002), with sessions from subjects of ethnicities likely to have higher hair occlusion (according to self-reported responses averaged by ethnicity) also having lower ROI quality scores (see Figure 9 left bottom). Note that this ordering sorts African Americans lower than Hispanics (also in agreement with the table reproduced in Maymone et al., 2021); reversing the order of these two bins yields a lower, but still-significant correlation (tau = −0.295, *p* = 0.025).

We then calculated the variance explained by each of these hair properties above contributing to ROI quality using an ANOVA, and found that taken together, they accounted for nearly 40% of total variance, with hair length, hair color, and hair density accounting for 16.1%, 14.2% and 8.5% of total variance, respectively (Table 4). We performed the same analysis using the available demographic factors for age, sex, and ethnicity (coded as unordered categories for the ANOVA). Here we find a total variance explained of nearly 60%, and 34.7%, 7%, and 16.8% for age, sex, and ethnicity, respectively. *P*-values are statistically significant (*p* < 0.01) for all factors after FDR multiple-comparison correction (Benjamini and Hochberg, 1995). A highly conservative Bonferroni multiple comparison correction across these comparisons show most factors retain significance except sex and hair density.

**Table 4:** Statistics for correlation comparison of signal quality vs. subject demographics and hair properties as shown in Figure 9 (top), and BCI performance vs. subject alertness and hair properties as shown in Figure 10 (bottom).

**ROI quality**	**p (raw)**	**p (FDR)**	**tau**	**rho**	**Wilcoxon**
Age	0.000	0.000 (^**^)	0.486	0.662	n/a
Sex	0.023	0.025 (^*^)	n/a	n/a	2.279
Ethnicity	0.001	0.002 (^**^)	−0.391	−0.562	n/a
Hair color	0.000	0.001 (^**^)	−0.399	−0.504	n/a
Hair density	0.000	0.001 (^**^)	−0.325	−0.402	n/a
Hair length	0.002	0.003 (^**^)	n/a	n/a	3.347
**Bci performance**	**p (raw)**	**p (FDR)**	**tau**	**rho**	**Wilcoxon**
Roi quality	0.004	0.006 (^**^)	0.412	0.579	n/a
Alertness	0.007	0.008 (^**^)	0.573	0.717	n/a
Hair color	0.087	0.087	−0.132	−0.181	n/a
Hair density	0.000	0.002 (^**^)	−0.296	−0.363	n/a
Hair length	0.001	0.003 (^**^)	n/a	n/a	3.073

Pearson's correlation coefficient, Kendall's tau, and Spearman's rho were calculated, and for binary quantities, a Wilcoxon rank-sum unpaired t-test statistic was used. The p-value and FDR-corrected p-value included here is of the Pearson's correlation coefficient. ^*^ and ^**^: Significance levels (thresholds as in Table 2).

#### 3.4.2 Block averages vs. hair and demographic factors

An attempt was made to directly correlate HRF block averages for HbO and HbR chromophores with the same battery of factors, however this proved challenging due to the high variability intrinsic to HRF responses in our data. No significant effects were found in this analysis, although we cannot rule out that better preprocessing would not reveal such associations. Note however, that both qualitative differences and differences in significant HRF effects were pronounced between the overall PQ and MVQ groups, as discussed in the Block averages section.

#### 3.4.3 BCI performance vs. quality, hair and demographics factors

**Figure 10:**
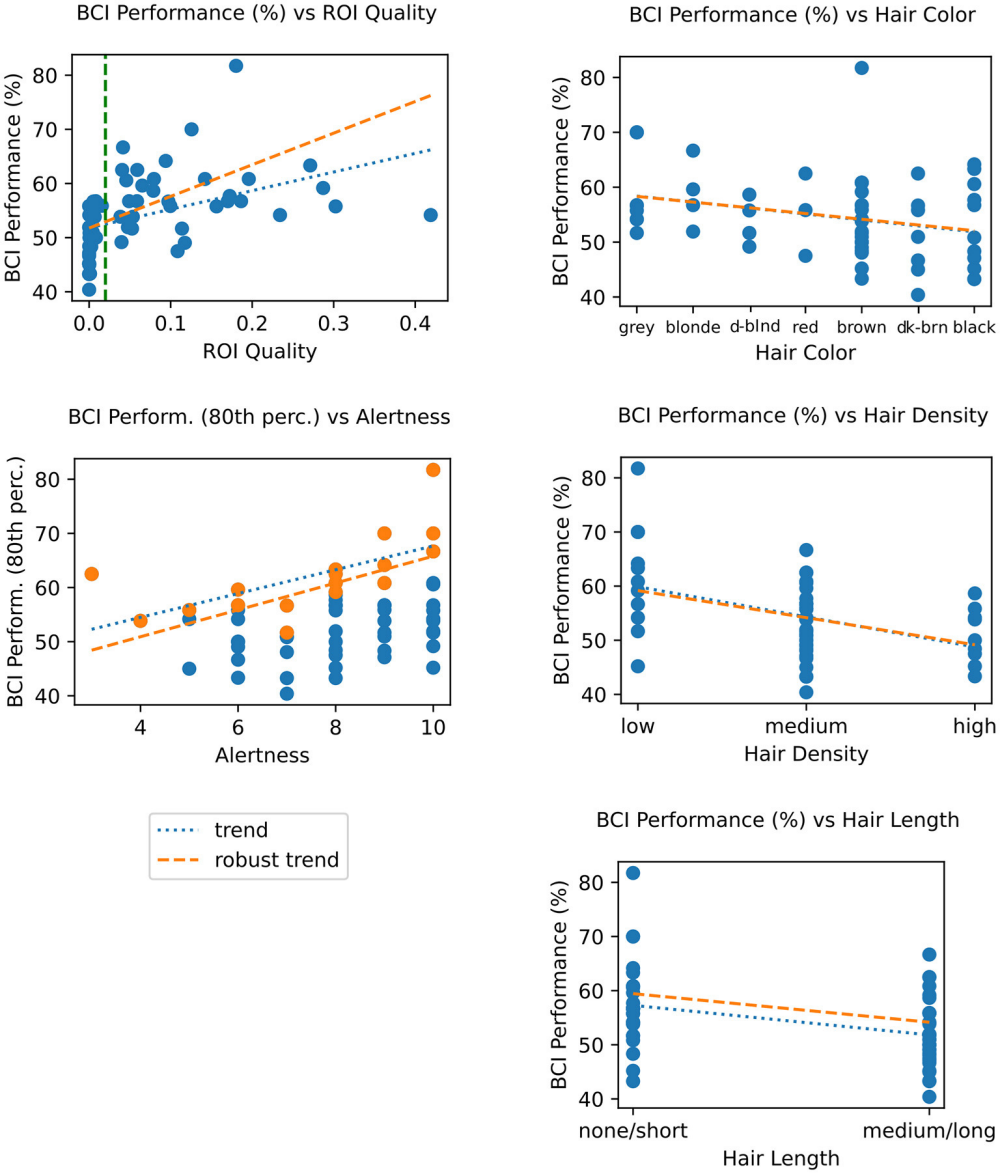
**Top left**: BCI performance (classification accuracy) vs. ROI Quality score; the green vertical line marks the threshold for binning sessions into “minimum viable quality” (MVQ) and “poor quality” (PQ) groups. **Bottom right**: BCI performance vs. alertness; dots highlighted in orange represent top 20% performers in each alertness bin; trends (fit on this subset) serve to illustrate hypothesized ceiling effect. **Right column**: BCI performance vs. hair properties, showing color **(top)**, density **(center)**, and length **(bottom)**. Other details as in Figure 9. For corresponding statistics, see Table 3.

We examined the correlation between the ROI quality score and BCI performance and found them to also be significantly correlated (tau = 0.412, *p* = 0.004) (shown in Figure 10 left top). Furthermore, we found that below *t*_qual_ (indicated by the dashed green line in the figure), BCI performance is spread around chance level for most sessions, with performance slowly increasing with better quality. This mirrors similar results between these two groups in terms of signal quality using a variety of metrics (Figure 4), and in neural responses (Figure 5).

Given the demonstrated correlation between quality and BCI performance, we then computed the direct relationship between the latter and hair factors contributing to quality, specifically hair color, density, and length. The results were similar to the trends and correlations found between those factors and ROI quality (Figure 10 right column). Specifically, hair density was significantly correlated (*r* = −0.363, tau = −0.296, *p* < 0.0005), as was (short/long) hair length (*T* = 3.073, *p* = 0.001, using a robust Wilcoxon rank-sum unpaired t-test). Hair color was not significantly correlated with BCI performance (*r* = −0.181); this was the main difference to the findings based on ROI quality.

Another factor that we assumed would be likely to impact BCI performance, though not signal quality, was the subjects' level of alertness, self-reported at the start of the session on a scale of 1 (Drowsy) to 10 (Alert). A direct comparison between performance and alertness level showed no significant correlation. Of note, however, we observed that these data appeared to show a “ceiling” effect, where the top-percentile scores across sessions binned by alertness appear limited by and correlated with alertness (Figure 10, right center, tau = 0.573). This was also not significant, likely due to the 5 times lower sample size in these top-performance sessions.

Finally, we computed the explained variance of BCI performance by hair characteristics, using a mass-univariate ANOVA. This is lower than the explained variance for ROI quality, with about 28% of the variance in BCI performance explained by hair characteristics (16.4%, 2.3%, and 9.8% for hair length, color, and density, respectively). The F-stat values were FDR corrected (Benjamini–Hochberg) and were statistically significant for hair length (binary categorization of none/short, and medium/long as in Figure 10, *p* < 0.001) and hair density (*p* = 0.007), but again not for hair color. Results are shown in Table 5.

**Table 5:** BCI performance variance explained by hair length, color and density, computed using a mass-univariate one-way ANOVA with FDR correction, along with the sum of squares, F-statistic and *p*-values.

**BCI performance**	**Variance explained**	**Sum of squares**	**F-statistic**	***p*-value (FDR)**
Hair length	16.37%	0.0576	13.0367	0.0006 (^**^)
Hair color	2.33%	0.0082	1.8549	0.1786
Hair density	9.75%	0.0343	7.7732	0.0072 (^**^)
Residual	71.55%	0.2517	n/a	n/a

^*^ and ^**^: Significance levels (thresholds as in Table 2).

## 4 Discussion

### 4.1 Related research

Our study employs a hand motor task based on sequential finger tapping that is modeled after tasks in which subjects tap fingers against a surface (here the desk), for instance Holper et al. (2009), Khan et al. (2010, 2012), and Nguyen et al. (2018). Throughout the article we analyzed exclusively data from motor imagery trials where this action is imagined, which comprised all the analyzed trials in our paradigm.

#### 4.1.1 Short-duration hand motor imagery tasks

Most fNIRS hand motor imagery studies utilize task durations of 10 to 20 s (e.g., Cui et al., 2010; Wriessnegger et al., 2018; Bak et al., 2019). Fewer studies attempt to use shorter durations which pose a greater challenge for BCI decoding due to higher single-trial variability, but may promise faster trial and bit rates. Fazli et al. (2012), Kaiser et al. (2014), and Chiarelli et al. (2018) employ 4, 5, and 6-second tasks, respectively, however all three are hybrid fNIRS/EEG studies. Two exceptions in fNIRS are Mihara et al. (2012) (5-s) and Peng et al. (2018) (7-s), the latter of which was a BCI study with continuous feedback. Similarly in motor execution paradigms, the majority of studies also utilize 10 to 20 s (or longer) trials, with few exceptions including Huppert et al. (2006) and Gagnon et al. (2012, 2014) (2, 2, and 5 s, respectively). Finger tapping, as studied here, is one of the most common types of hand motor studies in the literature (e.g., Sitaram et al., 2007; Holper and Wolf, 2011; Wu et al., 2018; Bak et al., 2019; Kwon et al., 2020), although we note that alternatives such as fist clenching, e.g., studied by Xu et al. (2014) or squeezing a ball or soft prop (e.g., Coyle et al., 2007; Batula et al., 2017) have also been used.

#### 4.1.2 Subject-dependent vs. subject-independent BCIs

Many fNIRS BCI studies to date (e.g., Holper and Wolf, 2011; Fazli et al., 2012; Xu et al., 2014) employ subject-dependent BCIs, i.e., trained on data from the subject in question, either from parts of the test session itself or from separate sessions of the same subject. The latter scenario avoids some issues in how preprocessing (e.g., whole-data statistics), trial selection (e.g., randomized cross-validation), or reduced cap fit variability within-session might lead to overestimating BCI performance relative to a full session-to-session transfer setting.

To date, only a small number of fNIRS studies have investigated entirely subject-independent BCIs, where no data from the target subject is needed to train or adapt the BCI. We choose this paradigm here among others to overcome the challenge of learning very high-dimensional models from relatively few fNIRS trials. To the best of our knowledge, the only example of such a study focusing on motor imagery is Fu et al. (2021), where cross-subject transfer of arm movement imagery BCIs was tested with a longer task duration of 8 s. That yielded a performance range of 60%−77% accuracy (no summary statistics were provided in this setting).

Outside motor imagery, three other examples are Kwon and Im (2021), where mental arithmetic vs. rest was tested (10 s. task), the work of Wang et al. (2021), where the current workload level in an n-back task was predicted (2 s. time windows of a long-duration task), and Trambaiolli et al. (2021), where the subject's binarized affective state was predicted (positive vs. negative or negative vs. neutral) from 30 s of data. Of these, Kwon and Im (2021) present, among others, results for a linear classifier (shrinkage LDA) and report an accuracy of 65.74% +/– 7.86% for that method. In Wang et al. (2021) the main focus was on hybrid calibration schemes where data from the target subject and other subjects was combined, although results for the pure subject-independent case were provided as well, where 55.6% accuracy was reported in a binary classification task, a near-chance result. Trambaiolli et al. (2021) reported statistically significant accuracies of 64.50 +/– 12.03% (positive vs. negative emotion) and 68.25 +/– 12.97% (negative vs. neutral emotion). In Kothe et al. (2023) we reported an accuracy of 67.7 +/– 10.4% when classifying low vs. high workload in an n-back task (*n* = 0 vs. 2, 40 s) across subjects in a similarly diverse population using the same high-channel decoding approach and using a different high-density fNIRS device.

#### 4.1.3 Data collection and analysis exclusion criteria

Due to the challenge of penetrating subjects' hair with NIRS optical probes (optodes), it is not uncommon to exclude subjects from analysis in current fNIRS studies, potentially at recruitment or screening time, at data collection time, or at analysis time, as has been discussed by, e.g., Khan et al. (2012) and more recently by Kwasa et al. (2023). With high-density devices, this problem can be exacerbated in a multitude of ways, for instance due to the time required to maneuver all optodes through the hair, or due to the inability to independently move closely spaced or rigidly mounted optodes.

However, as pointed out in Yücel et al. (2021), the practice of excluding subjects can inadvertently introduce systematic biases, since hair properties are correlated with, among others, age, sex, and ethnic background, and those factors in turn are strongly linked to differences in NIRS signal quality that can then drive data exclusion criteria. The sensitivity to hair factors has previously been demonstrated in Khan et al. (2012) and Fang et al. (2018) and acknowledged in Orihuela-Espina et al. (2010) and Scholkmann et al. (2014). However, literature exploring physiological and demographic confounds or biases due to such factors is otherwise relatively scarce, which prompted our choice to avoid any pre-biasing of the dataset at subject recruitment or data collection time.

### 4.2 ROI quality threshold and session partitioning

We found that when binning sessions into poor and minimum viable quality groups (PQ and MVQ) according to the proposed motor ROI-based quality threshold, the resulting partitioning also happened to separate sessions well according to quality observed in other channel locations, including the most distal channels. We infer this to be because quality observed at one scalp site is highly correlated with quality at another site if there are common underlying causes, such as specific hair and scalp properties.

The partitioning also helped separate PQ/MVQ session groups in our analysis of neural measures as further discussed in the below Section 4.3. The machine-learning/BCI analysis showed significantly above-chance accuracy for the MVQ group but chance-level accuracy for the PQ group. Moreover, the latter two analyses clearly demonstrate the consequences of pooling PQ and MVQ data (or equivalently, not rejecting sessions based on quality), namely that grand-average neural response estimates become significantly degraded and ML performance is likewise significantly impacted, averaging out at half-way between chance level and the MVQ group's average accuracy.

### 4.3 Neural measures

We found from block average analysis that HbO/HbR concentrations for the MVQ group present stereotypical canonical HRF waveforms, particularly for HbO, with most channels showing significant HbO peaks around 8 seconds after tapping onset. The finding of increased HbO agrees with previous fNIRS motor imagery studies that used longer imagined tapping durations [Holper and Wolf, 2011 (15 s), Mihara et al., 2012 (5 s), Iso et al., 2016 (20 s), and Batula et al., 2017 (20 s)], which also commonly show HbO (but not HbR) peaks around 8–12 s after onset for channels over the pre-motor and motor cortex areas. In contrast, the PQ group showed very noisy concentration waveforms with high variability across subjects and no significant time courses for any channels in either tapping condition. As might be expected, combining all sessions together produced roughly the average of both quality groups and showed relatively high variability across all channels and conditions, though there were several significant channels with HbO concentration peaks, whose latency is often extended out to 9–10 s after tapping onset. These results suggest that good-quality and plausible HRF waveforms can be obtained from block averaging of the 2 s tapping trials using our preprocessing chain of a bandpass filter, TDDR, and outlier removal *if* the relative signal quality is good, but HRF waveforms are greatly diminished when lower-quality sessions are included.

There remains a challenge in comparing the neural results of this study directly to previous literature as many fNIRS studies frequently use somewhat smaller sample sizes (< 20) and often report little detail regarding recruitment criteria with respect to demographics or hair phenotypes. As we have demonstrated here these factors can significantly impact signal quality which can affect the statistical results and conclusions made from neural analyses. Future fNIRS studies could benefit from even better understanding these factors and their impact on potential inferences and conclusions.

### 4.4 Machine learning analysis

Our original study aim of reaching high decoding accuracy on the collected fNIRS data was not achieved with any of the tested methods. Instead, we reached only modest BCI performance overall, even with the best tested method, and in analogy to the neural measures analysis, we identified a large difference in BCI performance between the two subject groups (PQ and MVQ). Specifically, a significantly above-chance accuracy of on average 59.1 +/– 6.7 was achieved when restricting cross-validation to the MVQ group, while a clear chance-level accuracy of 49.9 +/– 5.3% was obtained on the PQ sessions (even when including MVQ subjects in the training set), in both cases using a rigorous leave-one-subject-out cross-validation and the high-channel decoder. When including all subjects in the analysis, we obtained outcomes that were roughly in between those of the two data partitions, and in fact somewhat lower at 54.7 +/– 7.6%, which we attribute to the deleterious effects of including low-quality sessions in the training set. Nevertheless, at the same time these results represent one of the first subject-independent BCI results to date for fNIRS motor imagery data, and the first such analysis applied to short-duration motor tasks (here 2 s).

Due to the large available training set of over 30 sessions (in the MVQ set) and a high level of spatio-temporal regularization, we were nevertheless able to learn a high-resolution model, which is amenable to some amount of interpretation, including clear weight patterns compatible with hand motor activity of physiological origin during time windows corresponding to the imagined movements, possibly a signature of the initial dip, and evidence of implicit short-channel regression being performed by the model, all of which further support that the model leveraged patterns in good agreement with motor imagery decoding assumptions.

### 4.5 Factor analyses

The relatively large data sample and demographic metadata proved invaluable in our comprehensive study of potential causes of our signal quality, neural measures, and BCI results. Specifically, we performed an extensive analysis of associations between hair phenotypical and subject demographic factors vs. ROI quality scores (as defined in Section 2.4.), and ML performance. We reviewed each factor individually and tested for significant effects in dependent measures using both a robust and non-robust two-sample test (in the case of binary factors), or correlation test (for ordinal and continuous-valued factors), and reported the more conservative of the two.

We further performed FDR-corrected ANOVA analyses for groups of factors (separately for hair phenotype and demographic factors). These analyses showed significant effects of the following factors in outcome measures:

- Hair length (binarized as short vs. medium or long), hair color (coded as an ordinal factor by lightness), and hair density (self-reported 3-point scale, low/medium/high) each significantly influence ROI quality and collectively explain ca. 38.9% of the variance in ROI quality (*p* < 0.01 after FDR correction).
- Age, ethnicity (White, Hispanic, Black, Asian, in our sample), and sex (binary, in our sample) also each significantly influence ROI quality and collectively explain 58.6% of the variance in ROI quality (*p* < 0.01 after FDR correction).
- BCI performance was highly correlated with ROI quality (*p* < 0.005).
- Hair density and hair length each significantly influence BCI performance and collectively explain 26.1% of the variance in BCI performance (each *p* < 0.05 after FDR correction).
- Neither hair color nor alertness were found to be significantly correlated with BCI performance (although alertness appears to participate in an interaction effect).
- We attempted to construct quantitative measures of neural response effect size for inclusion in the correlation analysis, but these measures proved too noisy for analysis. No other tests yielding negative results were performed which are not mentioned or reported in this article.

Collectively, the factor analysis shows that effects of phenotypical and demographic factors on ROI quality are pervasive and highly significant. Furthermore, we showed that ROI quality itself is highly predictive of outcome measures such as BCI performance (accuracy) and neural responses (presence of significant effects), and hair phenotype is also directly predictive of BCI performance as would be expected on these grounds.

In this study, while 28 of the 61 sessions analyzed (46%) were from subjects with an ethnic origin other than “white,” once sessions were separated into MVQ and PQ groups using only the highly conservative ROI Quality threshold discussed earlier (Section 2.4.), only 26% of MVQ sessions (9 of 34) were from “non-white” subjects. This analysis leads to the conclusion that our results shown for the MVQ group, such as hemodynamic response curves, brain images, and ML model weights, should not be assumed to be representative of the unbiased general population, or of underrepresented ethnicities in particular. Due to the low quality of data obtained from the latter, we were not able to generate such results. More broadly, this situation underlines the importance of characterizing and accounting for potential demographic biases when data selection or exclusion criteria are employed that may in part be driven by hair phenotype or other demographics-linked factors.

### 4.6 Limitations and future work

While our results are striking and support the need for a deeper investigation of the nature of such biases along with applicable remedies, these results must nevertheless be interpreted in the context of the unique features and limitations of the present study:

1) We employ a lightweight wearable, LED-based headset with a high-density channel configuration. This alone contributes to individual channels having somewhat lower signal to noise ratios than what is found in many commercially available systems, due to factors including the shorter duty cycle of individual channels, the greater effort associated with working the many optodes through hair, and relative crowding of optodes and hair on the scalp. The headset also features a unique optode geometry using light guides that are relatively short (5 mm long) and which have 5 mm wide flat tips compared to some other point-tipped optode designs, or more elaborate interfaces such as, e.g., brush optodes (Khan et al., 2012). Also, optodes come in rigid assemblies (tiles) that do not allow for individual optode maneuverability. In combination, these circumstances limit the direct applicability of our findings to the somewhat narrow scope of headsets with similar probe designs, although the device can be viewed as state-of-the-art representative of a new generation of high-density headsets suitable for e.g., HD-DOT imaging and wearable/wireless setups.
2) High-channel/low-SNR configurations additionally pose a second challenge for BCIs, since higher-dimensional parameter vectors need to be estimated from limited training data, which is simultaneously less amenable to channel selection and sparse modeling due to low per-channel SNR, prompting the use of techniques such as cross-subject pooling and spatial smoothing to surmount these issues. A study that directly compares different types of fNIRS headsets, including low-density models, could shed light on designs and configurations that are optimally suited to minimizing these physiological biases.
3) The present study is not designed to replicate an idealized robust effect in a “textbook” setup involving generously long task times and, in the case of the BCI, ideal training data. Rather, we investigate here a purposely challenging setup where a well-known NIRS effect is pushed closer to the detection limit by use of a considerably shorter than commonly studied (and closer to potential real-world use cases) task performance time. Also, some of the presented results are for subject-independent BCIs, which suffer from additional factors of inter-subject variability.
4) Collecting more detailed data related to demographic characteristics, in particular hair characteristics such as length, thickness, and color, using objective numerical measurements rather than broader observation-based categories, would allow for more precise correlation measurements with signal quality and may improve sensitivity.
5) While the study was sufficient in size so as not to be underpowered, it would have benefited from more subjects having completed multiple sessions. This study was also designed to study BCI performance in the general population rather than to specifically compare between demographic groups, and therefore pseudorandom recruitment and almost no exclusion criteria was employed. A larger study without the constraints of data collection during COVID-19 and which actively recruits a weighted number of subjects across phenotypical and demographic categories could provide additional insights.

## 5 Conclusion

In summary, under the demanding circumstances of this study we observe a considerable impact of nearly every tested demographic and hair physiology factor on fNIRS (1) signal quality metrics, (2) neural responses, and (3) BCI accuracy. While the presented setting is certainly not representative of the typical fNIRS study, we nevertheless find that ethnographic biases including gender, ethnic origin, and age are potentially prone to be exacerbated near the detection limit and under conditions that may be increasingly relevant as fNIRS moves out of the lab and into real-world settings and populations. The results also renew the call for improved NIRS optode geometries and headset form factors that can overcome such challenges.

## Data availability statement

The raw data supporting the conclusions of this article will be made available by the authors, without undue reservation.

## Ethics statement

The studies involving humans were approved by UC San Diego Institutional Review Board, La Jolla, CA, United States. The studies were conducted in accordance with the local legislation and institutional requirements. The participants provided their written informed consent to participate in this study.

## Author contributions

CK: Conceptualization, Data curation, Formal analysis, Investigation, Methodology, Software, Validation, Writing – original draft, Writing – review & editing. GH: Conceptualization, Formal analysis, Investigation, Methodology, Validation, Visualization, Writing – original draft, Writing – review & editing. SM: Conceptualization, Data curation, Funding acquisition, Investigation, Methodology, Project administration, Supervision, Validation, Visualization, Writing – original draft, Writing – review & editing. TM: Conceptualization, Funding acquisition, Methodology, Project administration, Resources, Writing – original draft, Writing – review & editing.

## Footnotes

^1^ https://www.crowdsupply.com/starcat/hackeeg

^2^ https://www.crowdsupply.com/alaskit/hegduino-v2

^3^ https://store.neurosky.com/products/mindflex

^4^ https://choosemuse.com

^5^ https://www.gowerlabs.co.uk/lumo

^6^ https://www.neuropype.io

^7^ https://github.com/sccn/xdf

^8^ www.neuropype.io/

^9^This was done using scipy's resample function.
