# Supplementary material for: On decoding of rapid motor imagery in a diverse population using a high-density NIRS device
Source: Front Neuroergon. 2024 Mar 11;5:1355534. doi: 10.3389/fnrgo.2024.1355534 (PMC10961353; doi:10.3389/fnrgo.2024.1355534)
Supplement: Supplementary file 1 [file Data_Sheet_1.PDF]

Supplementary Material

1 Data Collection

1.1 Experiment Structure Summary

1.1.1 Room Setup / Subject Position

- The subject wearing the LUMO device sits in a room with low ambient light.
- The subject sits at a computer screen with reduced brightness. The subject rests their forearms on the armrests of an office chair and the desk in front of them so as to minimize any hand or arm movement during the task other than the tap action itself (when performed overtly only).
- When performing the tapping task, subject's palms are facing downward, wrist and fingertips resting on the table.

1.1.2 Task Instructions

- When prompted on screen, the subject performs the tapping task with either the right or the left hand, as indicated on screen, for a duration of 2 seconds.
  - Session1 (openloop): For most trials, tapping is covert (imagined motor movement). However a small number of overt (executed movement) trials are interspersed with the covert tasks at set times to reinforce the action that is to be made covertly.
  - Overt tap action: lift the index and middle finger of the same hand, in succession, and tap it against the table with moderate force. Repeat at a speed of about 1 Hz, so that each trial consists of 2 taps.
  - Covert tap action: rest all fingers resting on the table, and visualize performing the same tapping action described above, without moving the hand or fingers while intently focusing on each instance of the action. Mentally repeat each action at 1 Hz (two taps per trial).
- During the interstimulus intervals, the subject remains still.

During breaks, the subject can move/readjust, but remain seated. A message will appear 10 seconds before the end of the break to instruct the subject to remain still.

1.1.3 Experimental Conditions

Each session has equal numbers of left and right hand tap trials. All trials are of a 2 second duration. Total trials per session: Session1 (open-loop): 104 covert trials, 16 overt trials; Session2/Session3 (closed-loop): 120 covert trials.

1.1.4 Trial Structure

- Fixation Cross (5 seconds)
- Icon/text indication the action (tap, overt/covert, left/right) (2 seconds)

- Fixation Cross (6 seconds)
- Open-loop session: Fixation Cross cont'd (2 seconds)
- Closed-loop session: Feedback with BCI prediction (2 seconds) (closed-loop sessions only)

1.1.5 Block Structure

- Blocks are structured as a sequence of 30 trials of hand*duration conditions, in pseudorandom order: {Left, Right} x {2} seconds, yielding a total of 120 trials per session. Trial counts are balanced such that there is an equal number of trials per condition in the whole session, with no additional within-block balancing of trial types (so that the trial types do not become predictable to the subject towards the end of each block).
- Open-loop session: Each block is further broken down into 4 interleaved sub-blocks containing 13 covert trials and 2 overt trials, non-randomized, so that a 30 trial block sequence is: 13 covert, 2 overt, 13 covert, 2 overt. The purpose of the overt trials, used in openloop sessions only, is to reinforce the mental motion to be made as well as to add some variety to the task which can otherwise be monotonous without any feedback.
- Closed-loop session: Each block is broken down into 4 sub-blocks containing 15 covert trials.
- At the start of each sub-block, a screen appears with a mnemonic icon indicating both the action (tap) and mode (overt/covert) for the sub-block set, along with the number of trials in that set.
- The subject performs a practice block at the start of the session to become familiar with the task and instructions.
- One minute of baseline resting state data is collected after the practice block before starting the task blocks.

1.1.6 Session Structure

Description	Duration	Notes
Instructions		(see Instructions below)
Practice block	~8.5 min	
Resting state (baseline)	1 min	
Block 1	~8.5 min	30 trials, divided into 4 subblocks: 13 covert, 2 overt, 13 covert, 2 overt.
Break	0.5 min	10 sec before the end of the break, a screen appears instructing the subject to remain still for the remainder of the break (to avoid a delayed response at the end of the block affecting the first trial in the next block)
Block 2	~8.5 min	

Break	0.5 min	
Block 3	~8.5 min	
Break	0.5 min	
Block 4	~8.5 min	
Total duration	~50 min	

1.1.7 Stimuli and Cues

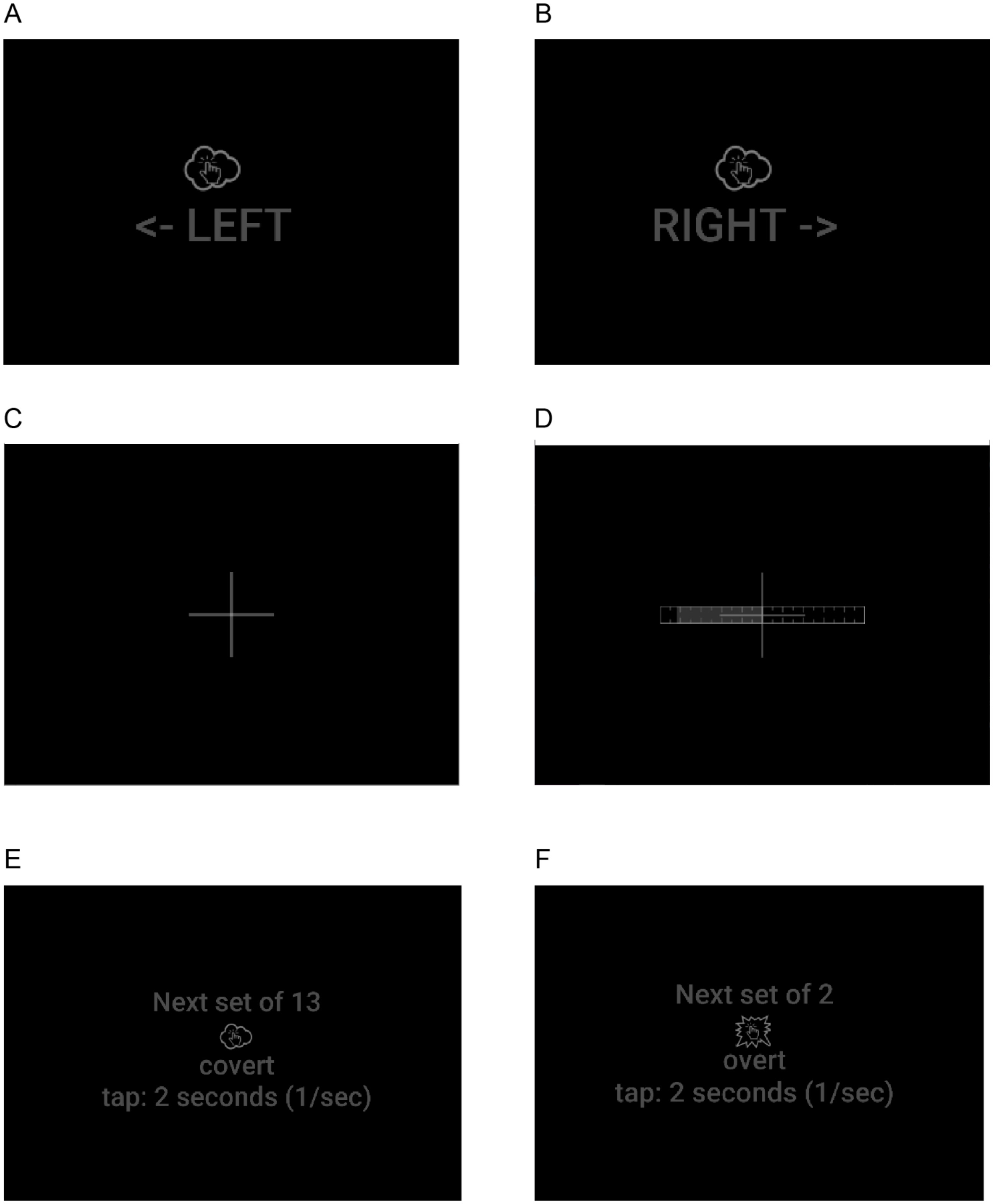

Supplementary Figure 1. Listing of stimulus screenshots used in the experiment. (A) Left-hand imagined tapping cue. (B) Right-hand imagined tapping cue. Executed (overt) trial variants use same mnemonic image as shown in screenshot (F). (C) Fixation cross shown pre prior to stimulus onset and following the task performance period. (D) BCI Feedback Gauge showing predicted

directionality (left/right) and degree of confidence. (E) Screen shown before each imagined/covert sub-block of here 13 successive trials (from Session 1). (F) As in (E), but for the executed/overt condition, here two successive trials (Session 1).

1.1.8 Instruction Text

The following instruction text is shown to the subject at the start of the session.

```
During this experiment you will perform a motor
imagery task.

A motor imagery task involves acting out the action
in your mind. Imagine sending the command to your body
to perform the action but stop short of any physical
movement (i.e, fingers or hand).

This is not the same as simply picturing yourself
doing the movement as if watching from a third-person
(or even first-person) perspective.

< page >

You will perform the following motor imagery task:
- imagined tapping of 2 fingers on a desk

Imagine tapping your index finger and then your
middle finger, in succession, with either the
right hand or the left hand.

For each trial (repetition), you will be shown
a word (LEFT or RIGHT) indicating the hand with
which you should imagine performing the action,
and for how long you should continue doing it.

Perform the imagined tapping for as long as you
see the word LEFT or RIGHT appear, stop when you
see the word disappear.

< page >

You should perform the action at the speed
of 1 tap per second.

Each trial (repetition) will last 2 seconds,
which means that for each trial you will imagine
tapping once with each finger.

< page >

Every so often you will be instructed
to physically perform the action ("overt") rather an
```

```
imagined movement ("covert"). This is to help
reinforce the feel of the action in your mind.

Before each set of trials you will be instructed
whether that set of trials is "overt" (physically
performed movement), or "covert" (imagined movement).

In between each trial, a fixation cross will
be shown, on which you should remain fixated
while remaining still.

< page >

< open-loop session only: >

Since this session is collecting data on which
to train a BCI model, the prediction of the BCI, which
would otherwise be shown at this stage, will not
be shown. Instead the fixation cross will continue
to be shown for the same period of time.

< closed-loop session only: >

During this time, the BCI will make a prediction
as to whether your imagined movement was with the
left hand or the right hand. Once the prediction
is computed, it will be displayed in the form of
a horizontal bar showing the direction of the
prediction (bar is left or right) and the degree
of confidence (how long the bar is).

Here you can see the how the BCI prediction
will be shown. In this example, the BCI predicted the
action was performed with the right hand
(with an approximately 80% confidence level).""")

<Graphic of BCI Feedback Gauge here>


< page >

There will be 4 blocks of 30 trials per block,
with a short break between each block.
Each block is further divided into sets of
13 "covert" trials and 2 "overt" trials.

The entire session will take approx. 40 minutes.
```

1.2 Subject Metadata

The following questions relevant to this study were included in the questionnaire filled out by subjects at the start of each session:

- Hair:
  - Strand thickness (fine, medium, thick)
  - Hair length (free text; operator separately indicated hair length as none, short, medium or long)
  - Hair density (low, medium, high)
  - Hair color (free text)
  - Haircut type (free text)
- Alertness (1 - 10 scale):
  - 1) Drowsy, 2) Fairly Drowsy, 3) Moderately Drowsy, 4) Somewhat Drowsy, 5) Slightly Drowsy, 6) Slightly Alert, 7) Somewhat Alert, 8) Moderately Alert, 9) Fairly Alert, 10) Alert
- Demographics:
  - Gender (male, female)
  - Ethnic Origin (free text)
  - Age (free text)
  - Handedness (right, left, ambidextrous)

2 Extended Block Average Results

The following figures list the additional block average results for different conditions and session subsets.

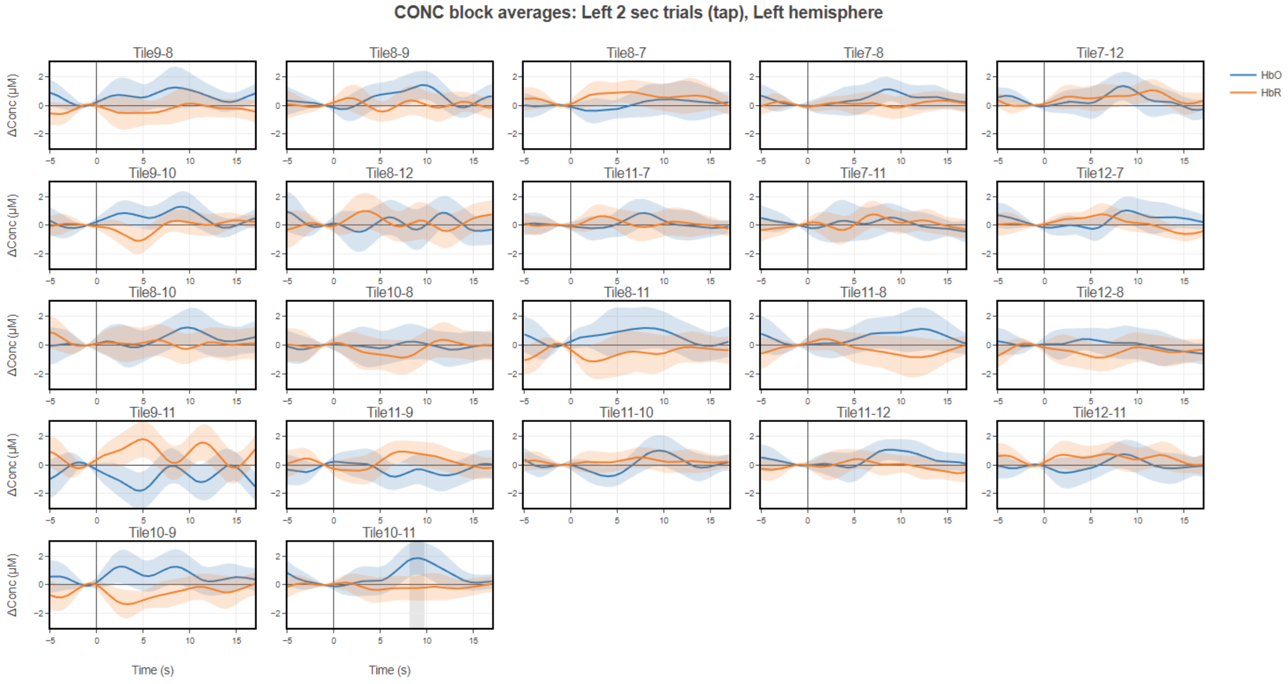

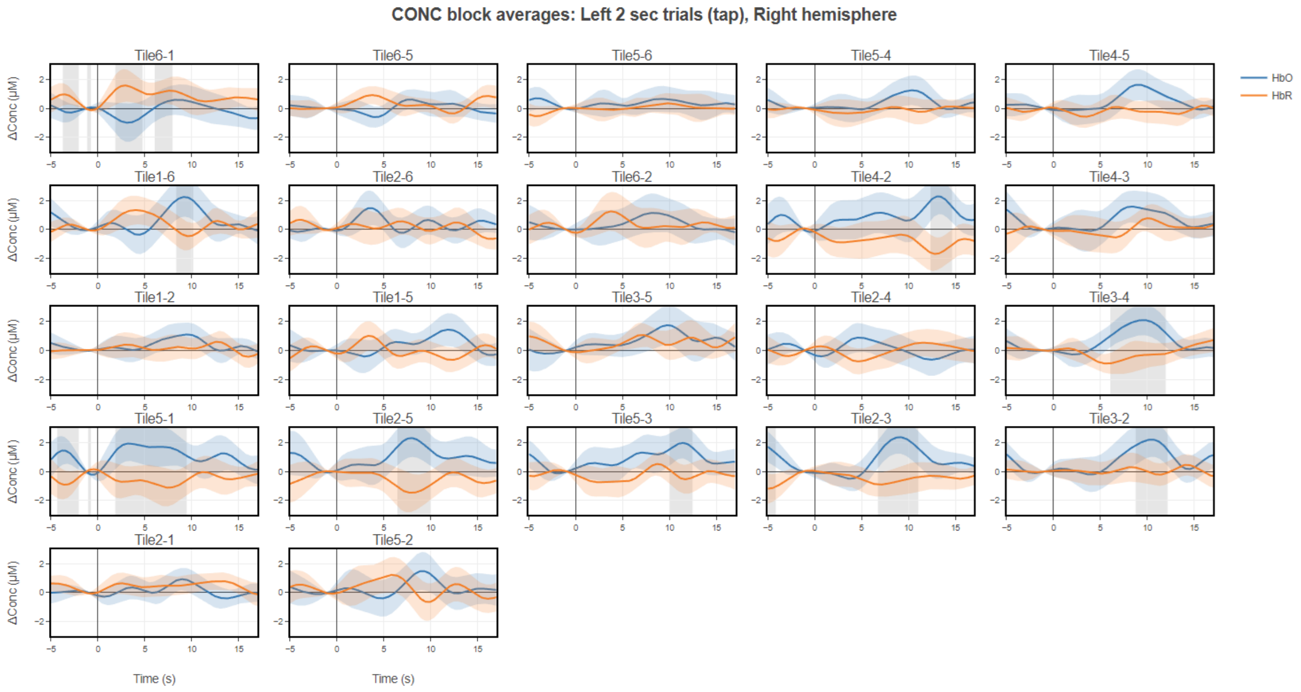

Supplementary Figure 2. All sessions mean block average concentrations of left and right hemispheres for 2 second imagery tapping (instructions presented at time point 0) with left hand for covert trials. Shaded error bars denote 95% confidence intervals. Gray bars indicate significant time periods ($p<0.05$, FDR corrected). Grid patterns of the channels for each hemisphere are laid out in relative anatomical locations such that the furthest right columns in the left hemisphere (and furthest left columns in the right hemisphere) are the most central over the brain.

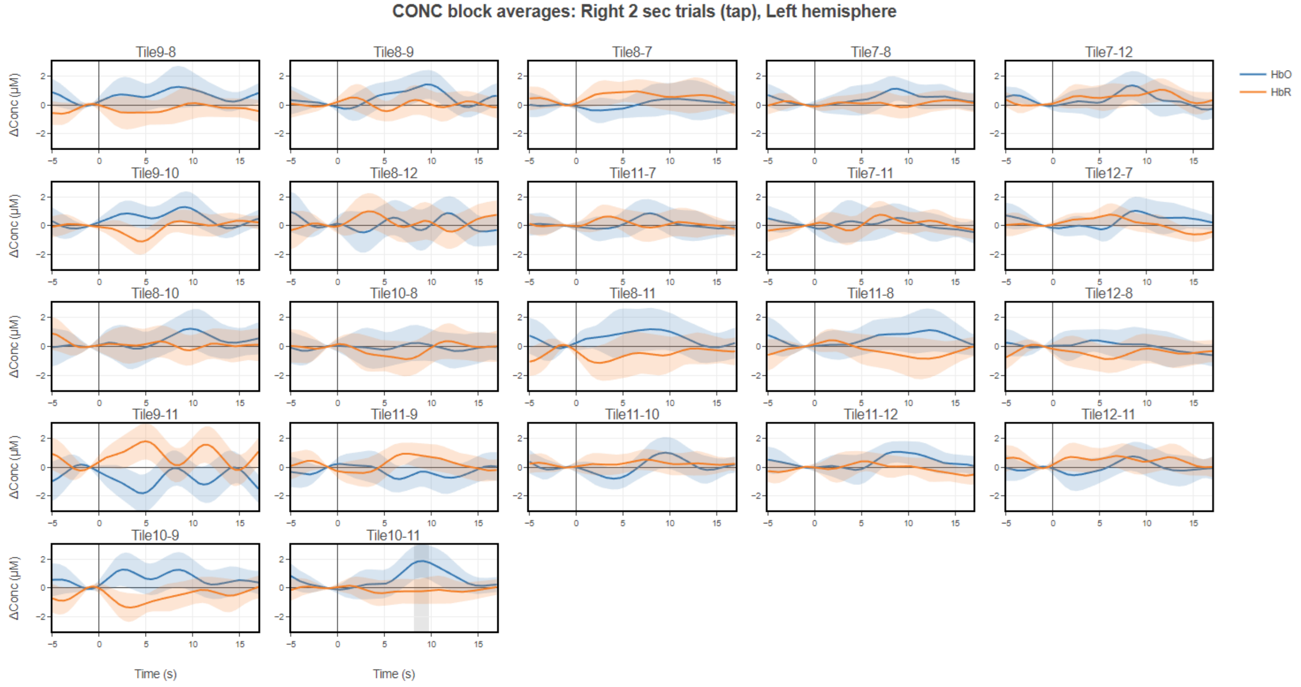

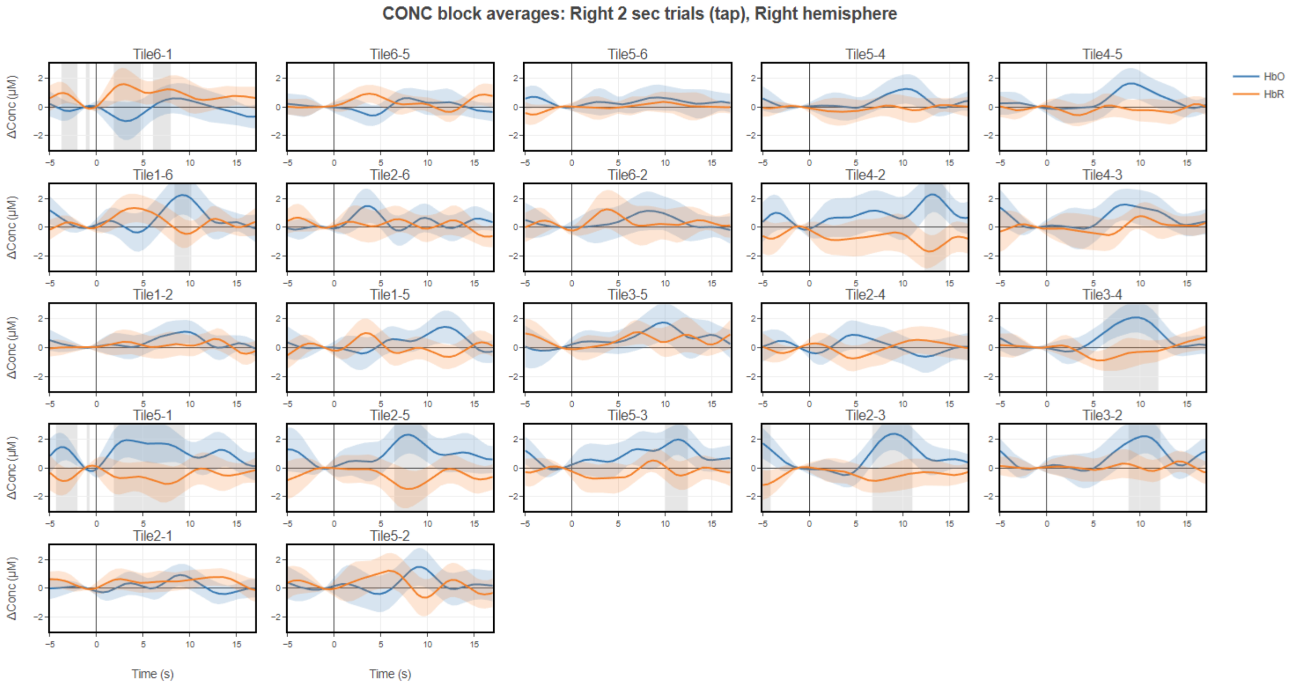

Supplementary Figure 3. All sessions mean block average concentrations of left and right hemispheres for 2 second imagery tapping (instructions presented at time point 0) with right hand for covert trials. Shaded error bars denote 95% confidence intervals. Gray bars indicate significant time periods ($p<0.05$, FDR corrected). Grid patterns of the channels for each hemisphere are laid out in relative anatomical locations such that the furthest right columns in the left hemisphere (and furthest left columns in the right hemisphere) are the most central over the brain.

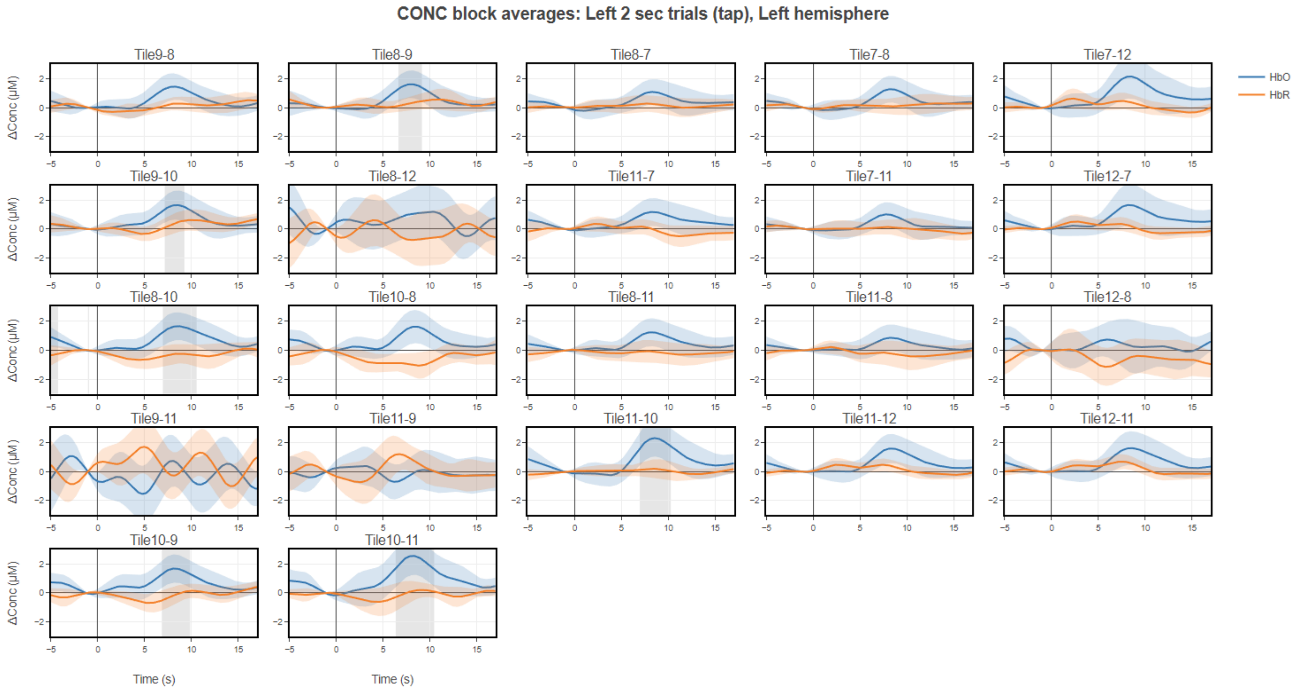

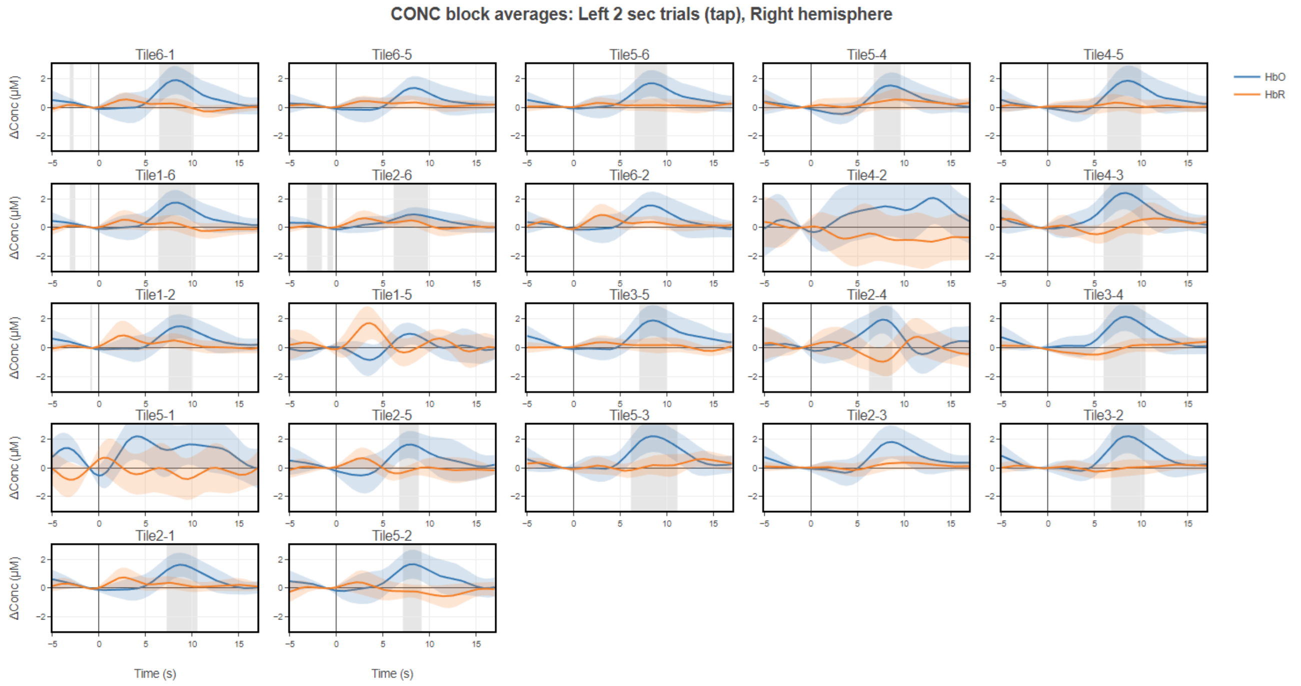

Supplementary Figure 4. Minimum Viable Quality (MVQ) sessions mean block average concentrations of left and right hemispheres for 2 second imagery tapping (instructions presented at time point 0) with left hand for covert trials. Shaded error bars denote 95% confidence intervals. Gray bars indicate significant time periods ($p<0.05$, FDR corrected). Grid patterns of the channels for each hemisphere are laid out in relative anatomical locations such that the furthest right columns in the left hemisphere (and furthest left columns in the right hemisphere) are the most central over the brain.

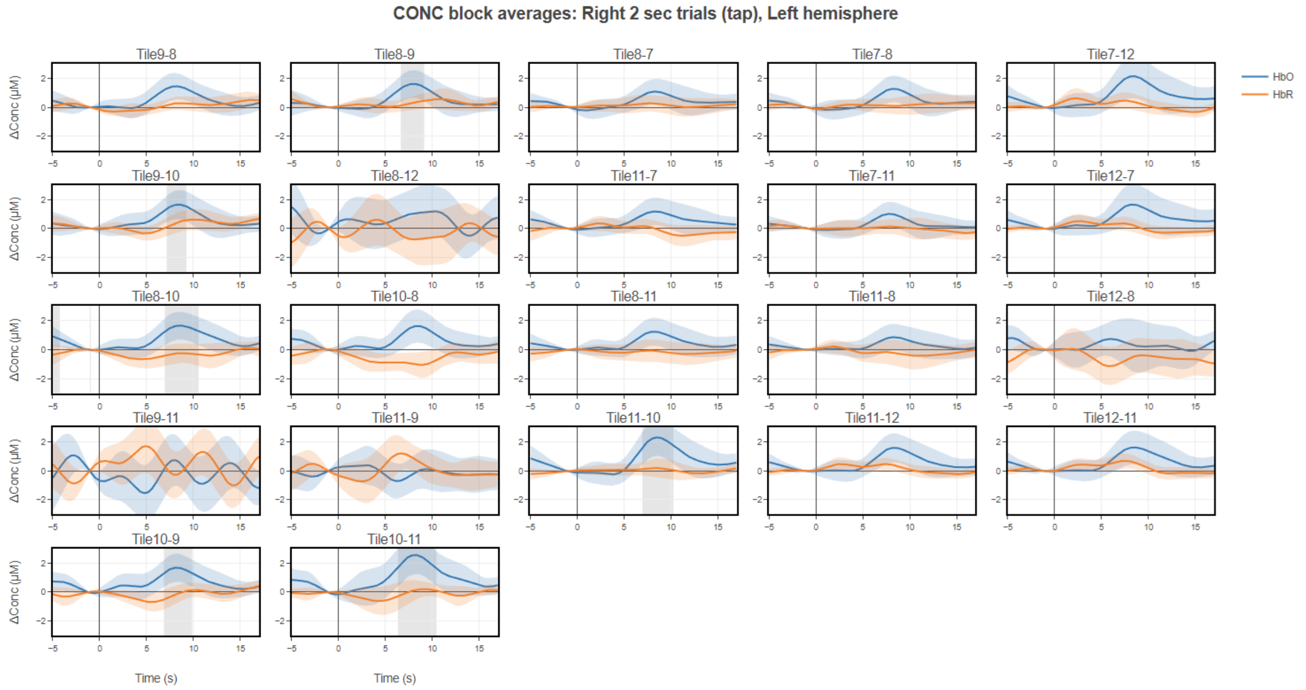

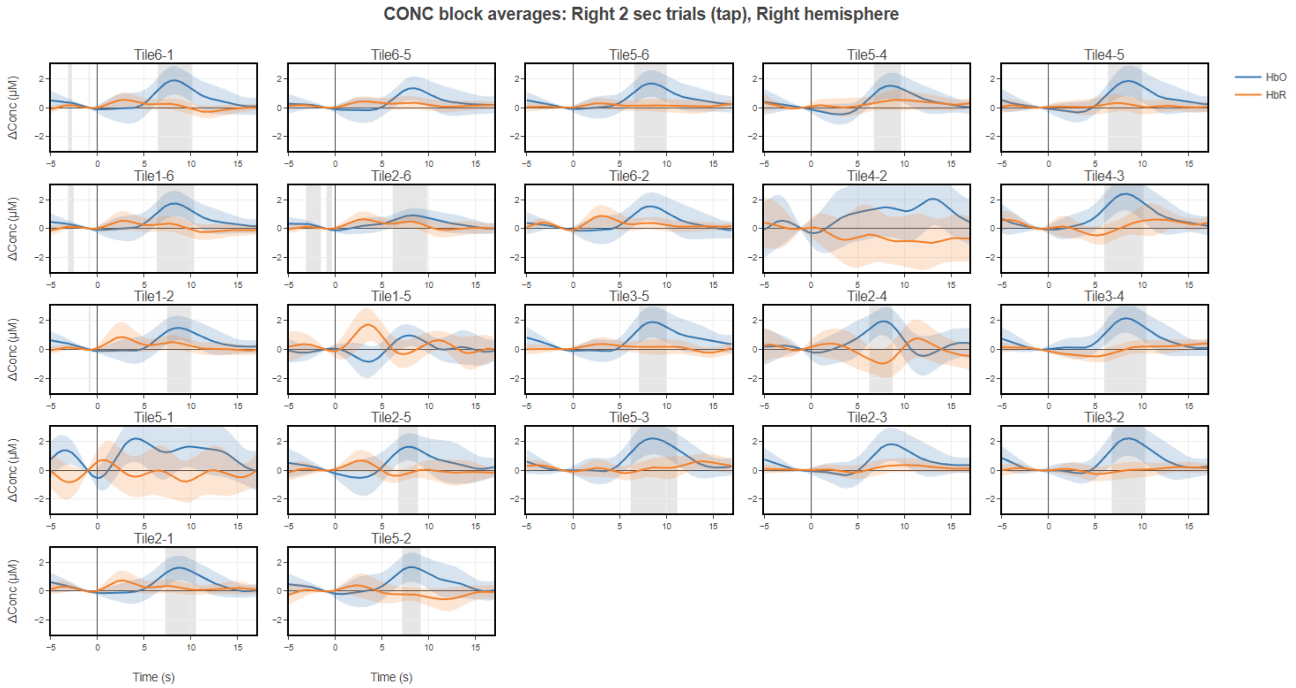

Supplementary Figure 5. Minimum Viable Quality (MVQ) sessions mean block average concentrations of left and right hemispheres for 2 second imagery tapping (instructions presented at time point 0) with right hand for covert trials. Shaded error bars denote 95% confidence intervals. Gray bars indicate significant time periods ($p<0.05$, FDR corrected). Grid patterns of the channels for each hemisphere are laid out in relative anatomical locations such that the furthest right columns in the left hemisphere (and furthest left columns in the right hemisphere) are the most central over the brain.

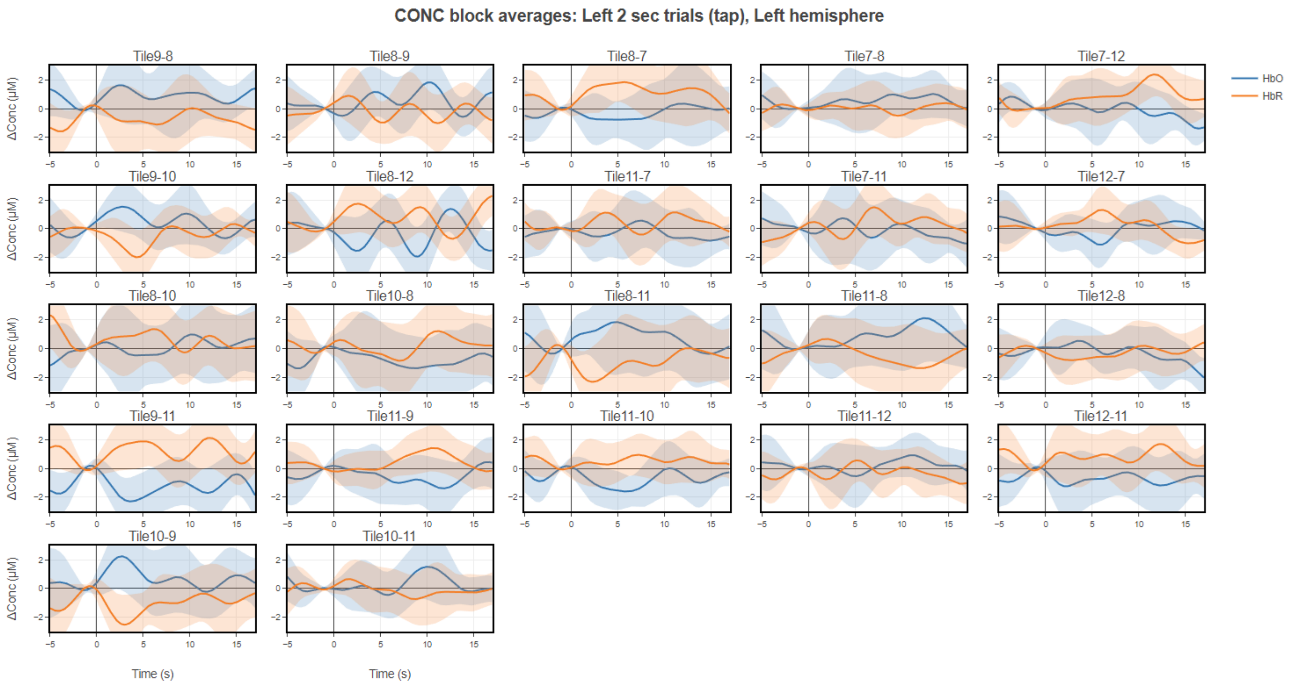

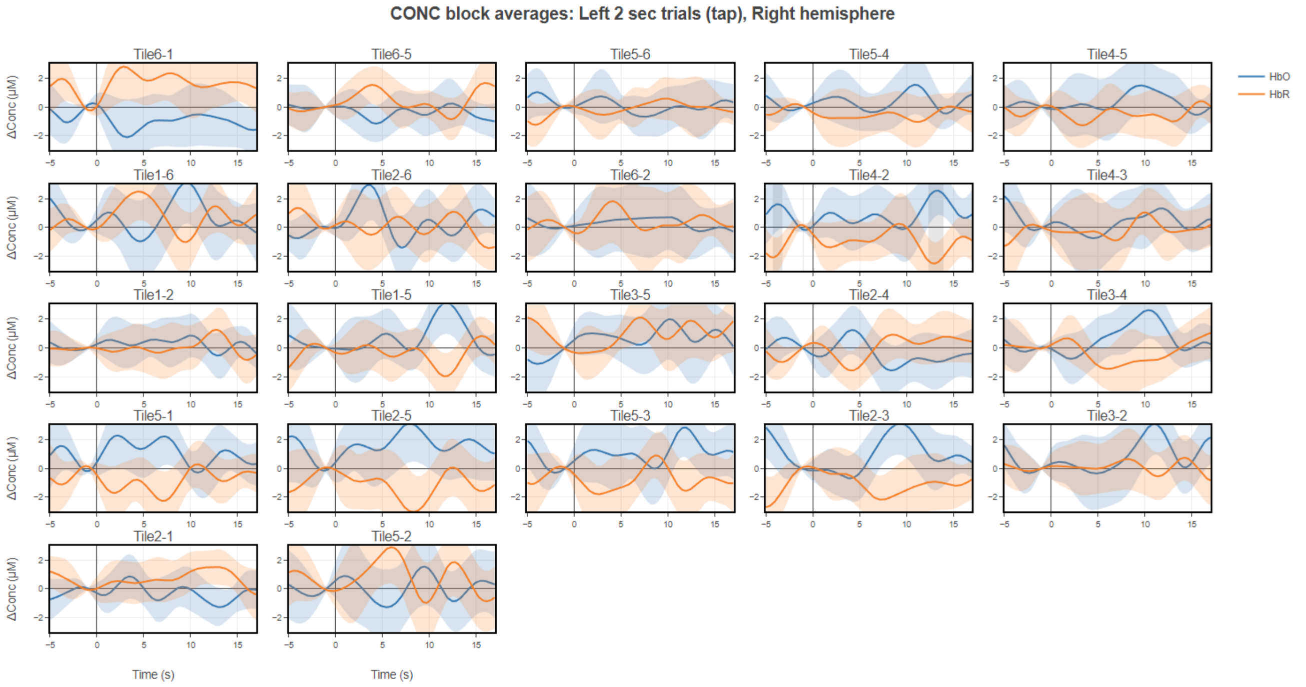

Supplementary Figure 6. Poor Quality (PQ) sessions mean block average concentrations of left and right hemispheres for 2 second imagery tapping (instructions presented at time point 0) with left hand for covert trials. Shaded error bars denote 95% confidence intervals. Gray bars indicate significant time periods ($p<0.05$, FDR corrected). Grid patterns of the channels for each hemisphere are laid out in relative anatomical locations such that the furthest right columns in the left hemisphere (and furthest left columns in the right hemisphere) are the most central over the brain.

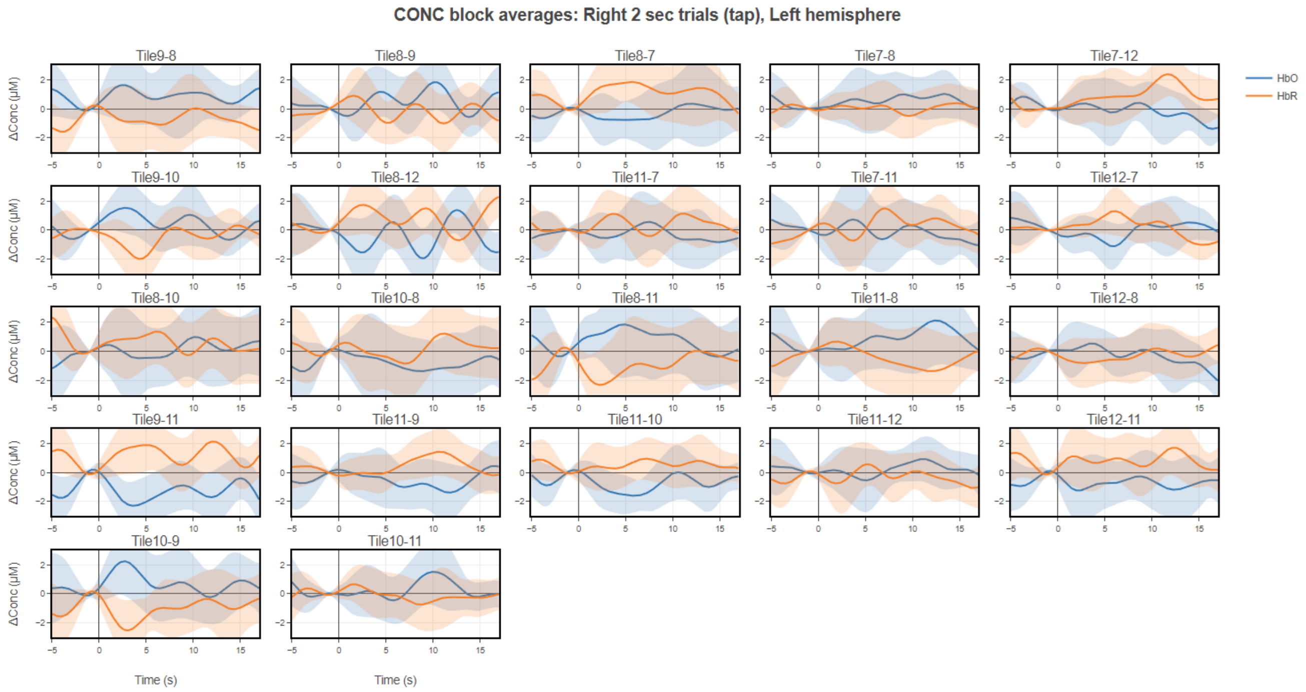

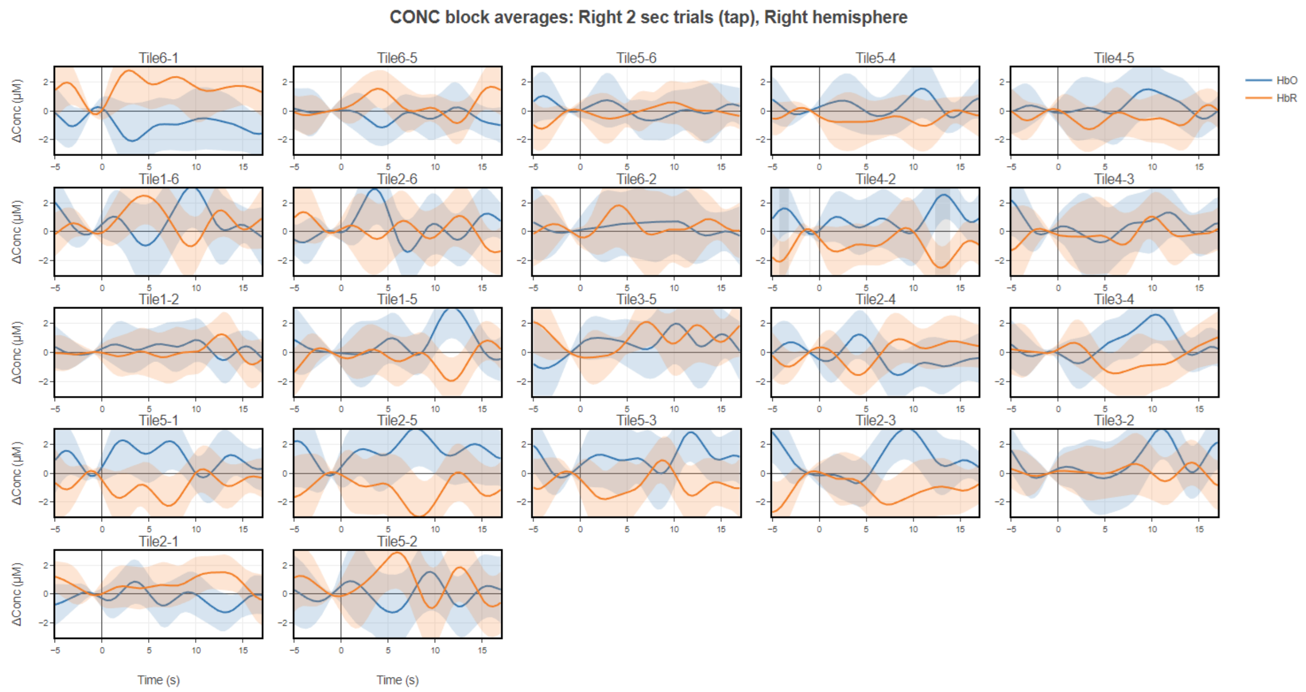

Supplementary Figure 7. Poor Quality (PQ) sessions mean block average concentrations of left and right hemispheres for 2 second imagery tapping (instructions presented at time point 0) with right hand for covert trials. Shaded error bars denote 95% confidence intervals. Gray bars indicate significant time periods (p<0.05, FDR corrected). Grid patterns of the channels for each hemisphere are laid out in relative anatomical locations such that the furthest right columns in the left hemisphere (and furthest left columns in the right hemisphere) are the most central over the brain.

3 ROI Quality Metric

In the following we provide a definition of the per-session ROI quality measure for a session *s* with respect to a set of ROIs $\mathcal{R}$ and a set of channels $\mathcal{C}$. This measure is a weighted coefficient of variation of all channels that intersect any of the desired regions of interest and is calculated as

$$Q_s(\mathcal{R}) = \sum_{c \in \mathcal{C}_L} \left(q_c w_c(\mathcal{R}) \Big/ \sum_{c \in \mathcal{C}_L} w_c(\mathcal{R}) \right)$$

where $q_c = 1 - 2\mathrm{CV}_c$ is a normalized quality score for a channel *c* based on its coefficient of variation CV_c, src_c and dct_c are the channel's 3d source and detector positions, and $\mathcal{C}_L = \{c \in \mathcal{C}\colon \|\mathrm{dct}_c - \mathrm{src}_c\| < L\}$ is the set of all channels of length no greater than *L* (here we set *L* = 50mm). The per-channel quality score q_c is then weighted according to its distance to the closest ROI in R that it intersects, according to the formula

$$w_c(\mathcal{R}) = 1 - S_n\left(\min_{r \in \mathcal{R}, t \in \{0..1\}} \|\mathrm{path}_c(t) - \mathrm{pos}_r\| / \mathrm{rad}_r\right)$$

where pos_r and rad_r are the center and radius of the ROI *r*, respectively. In our study we employ two ROIs, centered on the left- and right-hand motor cortex, respectively, and with a radius of 1cm

(centers were determined based on data of a prior motor imagery study). The function S_n is the n'th *smoothstep* polynomial (here *n*=4) that is used here as a radial falloff function, and the parametric curve

$$\mathrm{path}_c(t) = \mathrm{src}_c + t(\mathrm{dct}_\mathrm{c} - \mathrm{src}_c) + \mathrm{norm}_\mathrm{c}((2t-1)^2 - 1)\frac{\|\mathrm{dct}_\mathrm{c} - \mathrm{src}_c\|}{\tau}$$

is a quadratic approximation of the "banana"-shaped (e.g. Strangman et al., 2013) photon path for channel *c* where we denote by norm_c the scalp surface normal at the channel's midpoint. The constant τ is an assumed length-to-depth ratio of the photon path (we use τ=4 for the quality measure, therefore a channel of 4cm length would be assumed to have greatest sensitivity at a depth of 1cm below its linear midpoint). The above minimization can be performed by a grid search over *t*.

In the case of very high-density multi-distance NIRS data, this measure can be further improved by plugging in, instead of the raw per-channel measure q_c, a "denoised" estimate $\hat{q}_c$ that explicitly accounts for quality at the level of optodes. This measure has the effect of more explicitly factoring in the quality of the most relevant optodes for the ROIs of interest, rather than weighting strictly only the quality of the intersecting channels. We define this measure based on the quality for either the channel's source or detector optode, whichever is worse, i.e., $\hat{q}_c = \min\{\hat{q}_c(\mathrm{src}_\mathrm{c}), \hat{q}_c(\mathrm{dct}_c)\}$, where the score $\hat{q}_c(o) = 1/\left(1 + e^{\hat{w}_o\|\mathrm{dct}_\mathrm{c} - \mathrm{src}_c\| + \hat{b}_o}\right)$ describes the denoised quality of channel *c* with respect to optode *o* based on a per-optode falloff model of quality with increasing channel length. In line with the observation that q_c tends to fall off in a sigmoidal fashion with increasing length (see Fig. 3 (B) for a good example), we model the optode's quality falloff as a sigmoid parameterized by $\hat{w}_o$ and $\hat{b}_o$, which can be fit using either a logistic regression or a least-squares fit as in

$$\min_{w_o \in \mathbb{R}, b_o \in \mathbb{R}} \sum_{c \in \mathcal{C}_L, o \in \{\mathrm{src}_c, \mathrm{dct}_c\}} \left\| q_c - 1/\left(1 + e^{w_o\|\mathrm{dct}_\mathrm{c} - \mathrm{src}_c\| + b_o}\right) \right\|_2^2$$

We use the least-squares variant, which yields a non-convex problem in w_o, b_o that is however sufficiently well-behaved to be solvable with global optimization tools such as SHGO (Endres et al., 2018) with some sensible constraints (our analysis used $10^{-2} \leq w_o \leq 0.6$ and $-10^2 \leq b_o \leq 0$).

4 Real-time BCI used for feedback

The following summarizes the BCI that was originally used to generate feedback in Sessions 2 and 3. We separate the description into the prediction pipeline, which ran in real time, and the training pipeline, which was used to learn the weights of the prediction pipeline based on per-subject data.

4.1 Prediction Pipeline

The prediction pipeline was structured as follows:

- Retention of channels of at most 50mm length (for both the 56cm and 58cm cap sizes we assume the distances of the 56cm cap to ensure the same set of channels is retained)
- Optical density estimation (unreferenced, i.e., only log)
- 2nd order Elliptic IIR highpass filter (0.02-0.04 Hz transition band)

- 5-second sliding-window channel repair using a 0.25 correlation threshold and RANSAC-based robust interpolation
- Retention of channels whose photon path passes within at most 35mm of the left or right motor ROI
- HbO/HbR concentration estimation using the modified Beer-Lambert law (partial pathlength factor 0.1)
- 90-second sliding window standardization per channel
- Affine dimensionality reduction from retained channels to 300 spatial components (each dual-wavelength)
- Stimulus-locked segment extraction using the interval -1 to 10 seconds relative to stimulus onset (this yields a 10s decoding latency)
- Extraction of signal averages from successive time windows within the interval that follow a geometric progression from 200ms for the last window and successively earlier windows being 1.05x longer, yielding 27 temporal features per channel
- Apply learned feature scaling
- Linear model projection to logits (using logistic regression)
- Estimation of probabilities using the logistic sigmoid function

4.2 Training Pipeline

In summary, the training procedure for this pipeline operated as follows (steps that are analogous to the prediction pipeline use the same parameters, which are not repeated in full here).

- Estimation of grand average channel covariance matrix (per cap size):
  - For each subject
    - Retain channels < 50mm length
    - Calculate coefficient of variation (CoV) in 5s sliding window per channel
    - Flag windows with CoV/length in cm > 30 and mark windows as missing data
    - Calculate optical density (OD) and apply Elliptic IIR highpass filter, carry over missing-data mask
    - Calculate covariance matrix of OD in successive 10s windows
  - Calculate robust Huber centroid of covariance matrices with partial data
- Repair channels in a 5s sliding window (same as prediction pipeline)
- HbO/HbR concentration estimation using MBLL
- 90-second sliding window standardization
- Stimulus-locked segment extraction covering [-1s, 16s] relative to stimulus (whole ITI)
- Retention of channels within 35mm from left/right motor-cortex ROI centers (using shortest distance from photon path)
- Per-channel rescaling (z-scoring) using all per-session training epochs
- Reduction of trial/epoch segments to [-1s, 10s] interval
- Estimation of dimensionality reduction model across all subjects (not per cap)
  - For each class, across subjects:
    - Weight trial segments by raised-cosine window function (tail-heavy)
    - Estimate per-trial covariance matrices (uncentered, Dornhege et al. 2003).
    - Calculate robust Huber centroid of per-class covariance matrices
  - Find optimal spatial filters using Tikhonov Regularized CSP (Lotte and Guan 2010)

  - Retain best 300 components
- Apply component transform to segment data
- Extract geometrically spaced time-averaged features from trial segment
- Z-score each feature across all trials of session (The affine linear transform referred to in the prediction pipeline is the composition of dimensionality reduction followed by feature z-scoring)
- Learn logistic regression model with temporal smoothness regularization (l2 penalty) and spatio-temporal low-rank regularization (trace norm penalty)
